# Persistence and effectiveness of upadacitinib and TNFi for rheumatoid arthritis using pooled data from Canadian and Swiss registries

**DOI:** 10.1093/rheumatology/keag301

**Published:** 2026-06-23

**Authors:** Denis Choquette, Axel Finckh, Andrea Rubbert-Roth, Louis Coupal, Xiuying Li, Sibel Zehra Aydin, Helene Jonasch, Roger Gaertner, Dalinda Liazoghli, Mohammad Movahedi

**Affiliations:** Department of Rheumatology, CHUM, University of Montréal, Montréal, QC, Canada; Division of Rheumatology, Department of Medicine, Geneva University Hospital, Geneva, Switzerland; Geneva Centre for Inflammation Research (GCIR), University of Geneva, Geneva, Switzerland; Department of Rheumatology and Immunology, Cantonal Hospital St Gallen, St Gallen, Switzerland; RHUMADATA, Institut de Recherche en Rhumatologie de Montréal, Montréal, QC, Canada; Toronto General Hospital Research Institute, University Health Network, Toronto, ON, Canada; Division of Rheumatology, University of Ottawa, The Ottawa Hospital Research Institute, Ottawa, ON, Canada; AbbVie AG, Cham, Zug, Switzerland; AbbVie Corporation, Saint-Laurent, QC, Canada; AbbVie Corporation, Saint-Laurent, QC, Canada; Toronto General Hospital Research Institute, University Health Network, Toronto, ON, Canada; Institute of Health Policy, Management and Evaluation, University of Toronto, Toronto, ON, Canada

**Keywords:** upadacitinib, rheumatoid arthritis, registry, tumour necrosis factor inhibitor, persistence, effectiveness

## Abstract

**Objectives:**

The ROSA study evaluated real-world persistence and effectiveness of upadacitinib and tumour necrosis factor inhibitors (TNFi) in patients with rheumatoid arthritis (RA), using data from two Canadian registries, RHUMADATA and Ontario Best Practices Research Initiative (OBRI), and the Swiss Clinical Quality Management in Rheumatic Diseases (SCQM) registry.

**Methods:**

This observational study analysed data from 990 patients with RA who initiated upadacitinib or TNFi between 1 January 2020 and 30 June 2023. Multiple imputation was used for baseline covariates with missing data and results were adjusted for propensity scores derived from pre-specified variables (age, disease duration, prior treatments, comorbidities, etc.). Persistence was assessed using Kaplan–Meier and Cox proportional hazard regression models. Effectiveness was evaluated using observed (non-imputed) disease activity indices over 6 and 12 months.

**Results:**

Overall, 174, 216 and 600 patients were included from the RHUMADATA, OBRI, SCQM registries, respectively. Patients using upadacitinib had a lower risk of discontinuing treatment than patients using TNFi [hazard-ratio (HR) (95% CI): 0.602 (0.469, 0.773)] and this was unaffected by previous exposure to advanced treatment. Concomitant use of any csDMARD HR (95% CI); [upadacitinib: 0.225 (0.107, 0.471); TNFi: 0.516 (0.303, 0.881)], and methotrexate [upadacitinib: 0.604 (0.390, 0.934); TNFi: 0.694 (0.522, 0.925)] were associated with improved persistence for both groups. A similar proportion of patients receiving upadacitinib or TNFi achieved Clinical Disease Activity Index low disease activity or remission at 6 months (70.8% *vs* 61.0%; *P = *0.1541) and 12 months (70.5% *vs* 75.2%; *P = *0.4494).

**Conclusion:**

Canadian and Swiss patients with RA had longer persistence with upadacitinib than TNFi, independent of previous advanced treatments.

Rheumatology key messages

- Longer persistence for upadacitinib than TNFi in patients with rheumatoid arthritis in Canadian/Swiss registries.
- Longer persistence for upadacitinib versus TNFi regardless of previous exposure to advanced treatments.
- For patients with CDAI data, upadacitinib and TNFi had comparable effectiveness over 12 months.

## Introduction

Rheumatoid arthritis (RA) is a chronic inflammatory autoimmune disease characterized by the inflammation of synovial tissues. Inadequate treatment may result in joint damage, progressive disability and a reduced lifespan [1, 2]. Effective management of RA is crucial for alleviating pain and fatigue, improving quality of life and preventing further disability. The landscape of RA treatment has evolved significantly with the introduction of advanced therapies [3], including biologic (b)DMARDs like tumour necrosis factor inhibitors (TNFis) and non-TNFis, as well as targeted synthetic (ts)DMARDs such as Janus kinase inhibitors (JAKis) [2, 3]. One such advanced therapy is upadacitinib, an oral selective and reversible JAK-1 inhibitor (JAK1i) approved for the treatment of adult patients with moderately to severely active RA, either alone or in combination with methotrexate (MTX) or other conventional synthetic (cs)DMARDs [4]. Upadacitinib was approved for RA in Canada in December 2019 [5] and in Switzerland in January 2020 [6].

While randomized controlled trials (RCTs) are the cornerstone for regulatory approvals of new therapeutics, they are conducted in highly controlled environments with selective patient populations. Observational studies, however, offer valuable insights when multiple treatment options exist, and comparative RCTs are unavailable. These studies provide a broader perspective, as RCT populations may not fully represent the real-world population. Drug persistence in observational studies serves as a composite measure and index of effectiveness, tolerability and safety [7, 8]. Consequently, cohort-based registries are increasingly used to evaluate the effectiveness of therapeutic agents in routine clinical practice [9]. The efficacy and safety of upadacitinib to treat RA were evaluated in the SELECT phase 3 clinical trial program with six randomized trials.

Tumour necrosis factor inhibitors have a long history as a treatment for RA [10–12] and are often considered a first line of therapy. However, many patients with RA receiving TNFis do not achieve low disease activity or sustained remission [13]. Treatment persistence, which generally refers to the time from initiation to treatment discontinuation or switching to another therapy, is a critical surrogate marker for evaluating real-world clinical outcomes in RA management. A better understanding of treatment persistence is essential for enhancing patient prognoses and formulating informed therapeutic strategies in clinical practice.

To further investigate these real-world outcomes, we pooled data from two Canadian registries, RHUMADATA [14] and Ontario Best Practices Research Initiative (OBRI) [15], along with the Swiss Clinical Quality Management in Rheumatic Diseases (SCQM) registry [16], to describe the characteristics of patients with RA who have been initiated on either upadacitinib or TNFis. The approval dates of upadacitinib were similar and upadacitinib is accessible as first-line therapy in both countries. This approach provided a comparative analysis of the real-world persistence and effectiveness of these treatments in routine clinical practice, across countries with similar health care systems.

## Methods

### Study design and data sources

The ROSA observational study of prospectively collected data of patients with RA who initiated upadacitinib or TNFi treatment between 1 January 2020 and 30 June 2023. Data were collected from the RHUMADATA, OBRI and SCQM registries (Supplementary Table S1). The study described the persistence of upadacitinib and TNFis as a class of immunomodulatory drugs. Patients’ data were collected from treatment start until discontinuation, death, loss to follow-up or last visit, whichever came first. All definitive treatment cessations were considered. Time to treatment discontinuation was defined as (i) a gap of >30 days between upadacitinib dosing [17] and (ii) a gap of >90 days between TNFi dosing [18, 19], while a shorter gap was considered as treatment interruption, not discontinuation. These gap thresholds are supported by published methodological standards and reflect the different dosing schedules (upadacitinib is daily; TNFi, every 1–8 weeks), minimizing misclassification after a single missed dose, especially for TNFi. The discontinuation date was the date of the first missed dose. In this study, dosage change was not considered discontinuation. Each registry reported all treatment courses during the study period. For patients with multiple treatment courses, only the first course was included for the purpose of this study.

Patient identifiers were not required; anonymized, randomly assigned ID numbers were used for analyses.

This study was approved in Canada by the University Health Network Research Ethics Board (REB# 07–0729 AE) and in Switzerland by the Geneva ethical commission (CCER 2017–02228). The three registries also received ethics approval, and all patients provided written consent before their inclusion in the registries.

### Study population

This study included adult patients (≥18 years of age) diagnosed with RA, enrolled in one of the three registries who consented to have their data used for research. The start date was chosen to align with upadacitinib approval dates in Canada and Switzerland (19 December 2019 and 20 January 2020, respectively), mitigating selection bias by allowing for an equal chance of choosing upadacitinib or TNFis. Patients who did not consent to share their data with other researchers were excluded.

### Study objectives and endpoints

The primary objective of this analysis was to describe the real-world persistence of patients with RA who initiated upadacitinib or a TNFi. The secondary objectives included the comparison of drug persistence between upadacitinib and TNFi (i) overall, (ii) when used as first-line of advanced treatment or after having used at least one advanced treatment; and (iii) when used with a concomitant csDMARD or not. The comparison of effectiveness between upadacitinib and TNFi after 6 and 12 months of treatment was also evaluated as a secondary objective.

The primary end point was time to treatment discontinuation in patients using upadacitinib or TNFi. Secondary endpoints included time to treatment discontinuation for upadacitinib compared with TNFi overall and in various patient subpopulations (first-line therapy *vs* second or more line of advanced treatment, use of concomitant csDMARD) and measures of effectiveness at 6 and 12 months [Clinical Disease Activity Index (CDAI), Disease Activity Score 28 joint count C-Reactive Protein (DAS28-CRP, Health Assessment Questionnaire-Disability Index (HAQ-DI), pain, and patient global assessment of disease activity (PtGA)]. For all non-persistence secondary endpoints, data were analysed for patients treated for at least 6 and 12 months; therefore, for some patients enrolled later during the study, the observation period was extended up to 15 January 2024 to complete the observation period. For both the 6-month and 12-month time points, the visit closest to the target date within a ± 30-day window was selected. If multiple visits occurred within that window, the one nearest to the nominal time point was used.

### Statistical analysis

Statistical Analysis Software (SAS; version 9.4) was used for all analyses. Descriptive statistics for baseline (defined as start of treatment) characteristics were reported as mean and standard deviation (SD) for continuous variables and counts and proportions for categorical variables. Patients receiving upadacitinib and TNFi were compared using independent sample *t* tests for continuous variables and χ^2^ or Fisher’s exact tests for categorical variables. Time to treatment discontinuation for any reason was evaluated using Kaplan–Meier survival analysis (non-parametric model) and Cox proportional hazards regression analysis (semi-parametric models).

#### Multiple imputation, propensity scores

Multiple imputation was performed using the fully conditional specification method (FCS). A complete list of variables that were imputed, along with the proportion of available data for each, is provided in Supplementary Table S2. Different regression models were used to impute continuous and categorical variables. Twenty datasets were entered, and the results were combined using Rubin’s rules [20–22] (SAS Institute Inc. 2013. SAS/STAT^®^ 13.1 User’s Guide, Cary, NC, USA).

Treatment attribution is not random and could reflect patients’ specific factors. To balance predisposing factors that may increase a patient’s likelihood of receiving upadacitinib or TNFis, a propensity score was calculated for each patient using a set of pre-selected baseline covariates and variables found to differ between treatment groups at *P ≤ *0.1, as listed in Supplementary Tables S2 and S5. These included: age, disease duration, number of previous advanced treatments, data provenance, seropositivity (RF+ or ACPA+), comorbidities (hypertension, diabetes mellitus, anxiety/depression, lung disease), prior use of TNFi, prior use of a non-TNFi, prior use of JAKi, concomitant use of methotrexate, concomitant use of hydroxychloroquine, concomitant use of oral steroids, erythrocyte sedimentation rate (ESR), CRP, patient-reported pain, PtGA, physician global assessment of disease activity, tender and swollen joint counts, DAS28(4)ESR, HAQ-DI, and CDAI. The discontinuation rates were compared with Cox proportional hazard regression models applied on both unadjusted and propensity score-adjusted multiple imputation data, and the results were presented as hazard ratios (HR) and 95% CI.

Effectiveness outcomes were evaluated using observed (non-imputed) data only after baseline, as missing post-baseline disease activity might be due to treatment discontinuation or loss of follow-up and not imputed to avoid bias.

### Data access, handling and record keeping

Anonymized datasets were obtained from the OBRI and SCQM researchers via secured encrypted file transfer. The RHUMADATA research analyst created the pooled dataset by merging data from each registry to conduct analyses related to the protocol of this study only. No paper forms were exchanged between registries.

## Results

### Baseline characteristics

A total of 990 patients were included in this study across the three registries: RHUMADATA (174 patients); OBRI (216 patients); and SCQM (600 patients) (Table 1). The overall mean age at treatment initiation was 58.2 (SD: 13.4) years and the mean disease duration was 11.8 (SD: 9.5) years. At baseline, 551 (55.7%) patients had previously been treated with a TNFi, while 947 (95.7%) had used csDMARDs. Furthermore, 446 (45.1%) patients had prior use of an advanced treatment other than TNFis. Additionally, 238 out of 586 (40.6%) patients initiated TNFi as monotherapy, and 189 out of 404 (46.8%) patients started their upadacitinib as monotherapy (Table 2). Overall, the highest reported concomitant csDMARD treatment was MTX at 38.9%. In addition, 206 (20.8%) patients used concomitant oral steroids.

**Table 1:** Patient baseline characteristics.

	TNFi (N = 586)	Upadacitinib (N = 404)	*P* value	Total (N = 990)
Age at treatment initiation, years, mean ± SD (N)^a^	56.64 ± 13.98 (*N* = 586)	60.36 ± 12.12 (*N* = 404)	<0.0001^b^	58.16 ± 13.37 (*N* = 990)
Number of years of education, years, mean ± SD (N)	12.66 ± 2.00 (*N* = 478)	12.68 ± 2.31 (*N* = 336)	0.52^b^	12.67 ± 2.13 (*N* = 814)
Disease duration (from diagnosis to treatment initiation), years, mean ± SD (N)^a^	10.53 ± 9.04 (*N* = 574)	13.60 ± 9.85 (*N* = 394)	<0.0001^b^	11.78 ± 9.49 (*N* = 968)
Number of previous AT (biologics or others, excluding csDMARDs), mean ± SD (N)^a^	1.24 ± 1.63 (*N* = 586)	2.68 ± 2.16 (*N* = 404)	<0.0001^b^	1.83 ± 1.99 (*N* = 990)
Data provenance, *n* (%)			<0.0001^c^	
RHUMADATA^a^	85 (14.5%)	89 (22.0%)		174 (17.6%)
OBRI	108 (18.4%)	108 (26.7%)		216 (21.8%)
SCQM	393 (67.1%)	207 (51.2%)		600 (60.6%)
Women, *n* (%)	442 (75.4%)	329 (81.4%)		771 (77.9%)
Smoking status [non-smoker, ever smoked (i.e., smoker or ex-smoker)], *n* (%)			0.99^c^	
Never	234 (48.2%)	171 (48.3%)		405 (48.3%)
Ever	251 (51.8%)	183 (51.7%)		434 (51.7%)
RF+, *n* (%)	176 (59.7%)	148 (61.4%)	0.68^c^	324 (60.4%)
ACPA+, *n* (%)	126 (56.0%)	115 (62.8%)	0.16^c^	241 (59.1%)
Seropositive (RF+ or ACPA+)^a^, *n* (%)	196 (65.6%)	173 (70.6%)	0.21^c^	369 (67.8%)
Comorbidities present at treatment initiation, *n* (%)				
Hypertension^a^	120 (24.4%)	118 (31.9%)	0.015^c^	238 (27.6%)
Cardiovascular disease	59 (13.0%)	49 (14.8%)	0.47^c^	108 (13.7%)
Diabetes mellitus^a^	41 (8.8%)	49 (14.8%)	0.0093^c^	90 (11.3%)
Anxiety/depression^a^	77 (15.3%)	78 (21.6%)	0.017^c^	155 (17.9%)
Lung disease^a^	76 (16.0%)	74 (21.4%)	0.047^c^	150 (18.2%)
Gastrointestinal disease	118 (23.6%)	90 (25.7%)	0.49^c^	208 (24.5%)
Cancer	54 (10.3%)	40 (10.7%)	0.85^c^	94 (10.4%)
Prior use of csDMARDs (MTX, HCQ, SSZ and LEF), **n** (%)	560 (95.6%)	387 (95.8%)	0.86^c^	947 (95.7%)
Line of AT^a^, **n** (%)			<0.0001^c^	
First line AT	260 (44.4%)	66 (16.3%)		326 (32.9%)
Second line AT	134 (22.9%)	75 (18.6%)		209 (21.1%)
Third line AT or more	192 (32.8%)	263 (65.1%)		455 (46.0%)
Prior use of a TNFi^a^, **n** (%)	276 (47.1%)	275 (68.1%)	<0.0001^c^	551 (55.7%)
Prior use of an AT other than a TNFi^a^, **n** (%)	185 (31.6%)	261 (64.6%)	<0.0001^c^	446 (45.1%)
Prior use of a JAKi^a^, **n** (%)	126 (21.5%)	204 (50.5%)	<0.0001^c^	330 (33.3%)
Concomitant csDMARDs, **n** (%)				
MTX^a^	244 (41.6%)	141 (35.0%)	0.035^c^	385 (38.9%)
HCQ^a^	70 (12.0%)	64 (15.8%)	0.080^c^	134 (13.5%)
SSZ	39 (6.7%)	25 (6.2%)	0.77^c^	64 (6.5%)
LEF	74 (12.6%)	38 (9.4%)	0.12^c^	112 (11.3%)
Concomitant use of NSAIDs, **n** (%)	138 (47.9%)	117 (45.7%)	0.61^c^	255 (46.9%)
Concomitant use of oral steroids^a^, **n** (%)	105 (17.9%)	101 (25.0%)	0.0070^c^	206 (20.8%)

a Indicates that the variable was used to derive the propensity score (pre-selected variables or *P* value <0.1). *P* values are based on the ^b^Mann–Whitney test and the ^c^χ^2^ test.

cs: conventional synthetic; HCQ: hydroxychloroquine; JAKi: Janus kinase inhibitor; LEF: leflunomide; MTX: methotrexate; OBRI: Ontario Best Practices Research Initiative; SCQM: Swiss Clinical Quality Management in Rheumatic Diseases; SSZ: sulfasalazine; TNFi: tumour necrosis factor inhibitor.

**Table 2:** Treatment and cessation.

	TNFi (N = 586)	Upadacitinib (N = 404)	*P* value	Total (N = 990)
Combination therapy with csDMARD, *n* (%)	348 (59.4%)	215 (53.2%)	0.0450^c^	563 (56.9%)
Treatment duration (years), mean ± SD				
All patients	1.2517 ± 0.9642	1.4661 ± 0.9919	0.0007^b^	1.3391 ± 0.9808
Monotherapy	1.1387 ± 0.9135 (*N* = 238)	1.2631 ± 0.9573 (*N* = 189)	0.1718^b^	1.1938 ± 0.9341 (*N* = 427)
Combination therapy	1.3289 ± 0.9914 (*N* = 348)	1.6444 ± 0.9897 (*N* = 215)	0.0003^b^	1.4494 ± 1.0017 (*N* = 563)
*P*-value mono *vs* combo^b^	0.0189	0.0001		<0.0001
Treatment stopped, *n* (%)	223 (38.1%)	110 (27.2%)	0.0004^a^	333 (33.6%)
Reason for treatment cessation,*n* (%)			0.091^a^	
Ineffectiveness	127 (57.0%)	53 (48.2%)		180 (54.1%)
Adverse events	39 (17.5%)	33 (30.0%)		72 (21.6%)
Other^c^	57 (25.6%)	24 (21.8%)		81 (24.3%)

*P* values are based on the ^c^χ^2^ test and the ^c^*t* test.

c Includes reproductive issues, improvement, patient decision, medical decision, physician decision, unspecified and lost to follow-up.

cs: conventional synthetic; TNFi: tumour necrosis factor inhibitor.

A similar number of Canadian and Swiss patients initiated upadacitinib treatment [197 (48.8%) and 207 (51.2%), respectively], whereas more patients from Switzerland started a TNFi than patients from Canada [393 (67.1%) *vs* 193 (32.9%), respectively]. Patients who started upadacitinib were significantly older (60.4 *vs* 56.6 years, *P < *0.0001), had a longer disease duration (13.6 *vs* 10.5 years, *P < *0.0001) and had a higher mean number of previous advanced treatments (2.7 *vs* 1.2, *P < *0.0001) than patients starting a TNFi. Patients treated with upadacitinib had a higher proportion of comorbid hypertension (31.9% *vs* 24.4%; *P = *0.015), diabetes mellitus (14.8% *vs* 8.8%; *P = *0.0093), anxiety/depression (21.6% *vs* 15.3%; *P = *0.017) and lung disease (21.4% *vs* 16.0%, *P = *0.047) compared with TNFi. The proportion of patients with prior use of an advanced treatment at baseline was higher in patients receiving upadacitinib compared with TNFis (83.7% *vs* 55.6%; *P* *<* 0.0001), as was prior use of a TNFi (68.1% *vs* 47.1%; *P < *0.0001) and prior use of a JAKi (50.5% *vs* 21.5%; *P < *0.0001). With respect to concomitant csDMARDs, a higher proportion of patients receiving TNFi were receiving MTX compared with upadacitinib (41.6% *vs* 35.0%; *P = *0.035).

### Drug persistence in patients who initiated upadacitinib or TNFi

The overall mean treatment duration was 1.34 years (SD: 0.98) or 16.1 months, with 563 (56.9%) patients receiving combination therapy at baseline (Table 2). During the study, 333 patients (33.6%) discontinued their treatment. Among these, the most common reasons for stopping were ineffectiveness (54.1%, 180 patients) and adverse events (21.6%, 72 patients). A quarter of the patients discontinued their treatment within 0.72 years (95% CI: 0.60, 0.90) (8.6 months) of treatment, while 50% of the patients stopped by 3.5 years (95% CI: 3.12, not estimable).

A Cox regression analysis showed that patients using upadacitinib had a lower risk of discontinuing treatment than patients using a TNFi (HR: 0.602; 95% CI: 0.469, 0.773; *P < *0.0001) (Supplementary Table S3). Combination therapy (HR: 0.730; 95% CI: 0.585, 0.912; *P = *0.0055) and disease duration (HR: 0.980; 95% CI: 0.967, 0.994; *P = *0.0041) were also associated with lower risk of discontinuation and each previous advanced treatment was associated with an increased risk of discontinuation (HR: 1.111; 95% CI: 1.048, 1.177; *P = *0.0004). Similar results were obtained when data were adjusted with propensity score. Also, several Cox regression models were employed to analyse the dataset without missing values with similar results (Supplementary Table S4).

### Comparison of drug persistence between upadacitinib and TNFi

**Figure 1:**
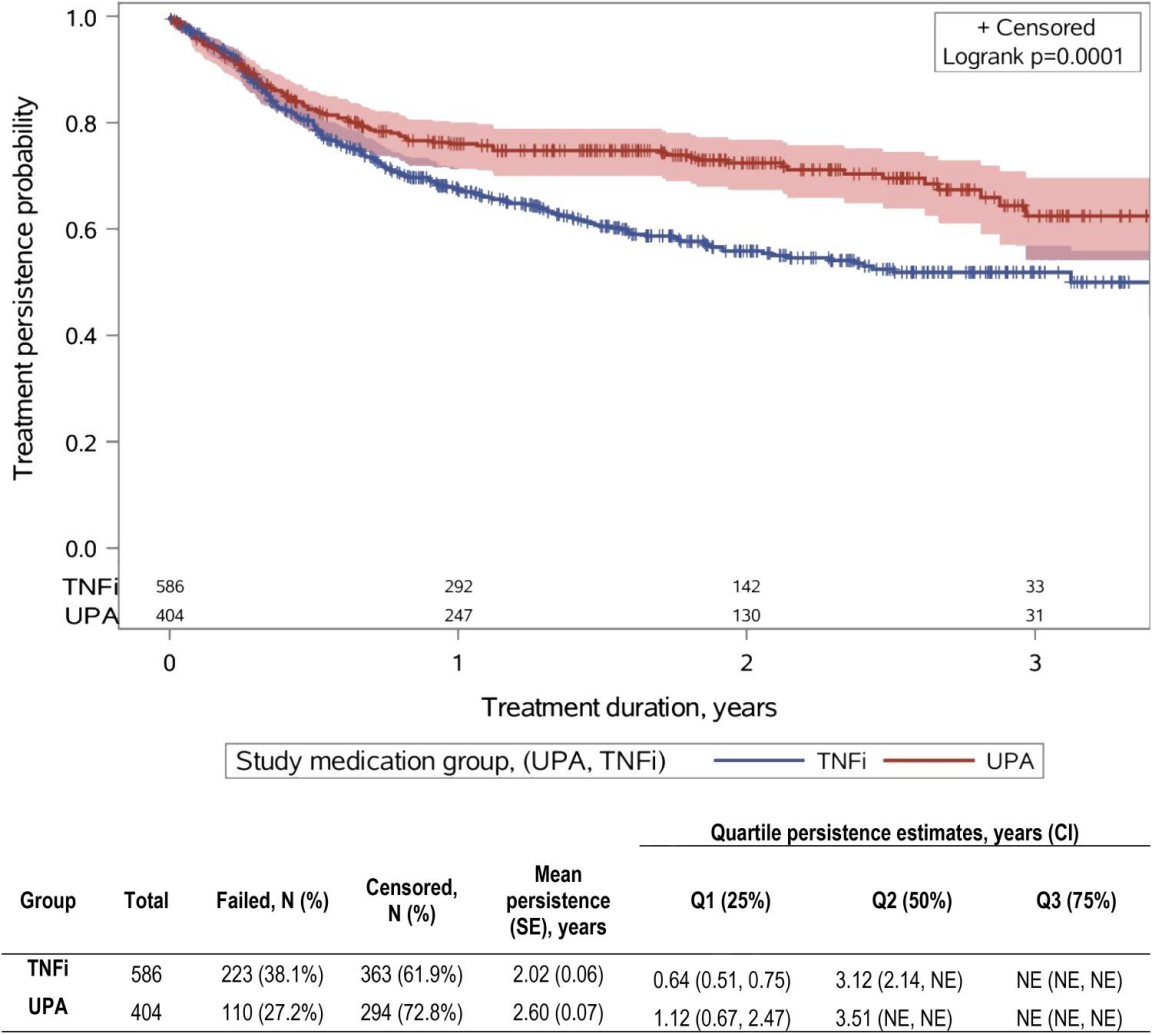
Comparison of time to treatment discontinuation between TNFi and upadacitinib. Only the first episode of patients with multiple episodes during the study period was kept. Shaded area represents 95% CI. NE, not estimable; SE, standard error; TNFi, tumour necrosis factor inhibitor; UPA, upadacitinib

Patients treated with upadacitinib had a significantly longer mean treatment duration compared with those receiving a TNFi (1.47 *vs* 1.25 years; *P = *0.0007, Table 2). A quarter of the patients (25%) using TNFi had discontinued treatment by 0.64 years (7.7 months) compared with 1.12 years (13.4 months) for patients using upadacitinib. The overall drug persistence of upadacitinib was significantly longer than TNFis, as analysed using Kaplan–Meier estimates (log-rank *P = *0.0001) (Fig. 1). Persistence was also assessed across different lines of advanced treatment, with significant differences between upadacitinib and TNFi used as first-line (*P = *0.0027); second-line (*P = *0.0103); third-line or more (*P = *0.0191) including after having used medications with >1 mode of action before initiation the current treatment regimen (*P = *0.0230; Supplementary Fig. S1). Notably, patients using upadacitinib as first line of advanced treatment had significantly higher persistence that patients using upadacitinib as third line or more of advanced treatment (long-rank *P = *0.0319, Supplementary Fig. S2). Of note, additional analyses have shown variation in persistence patterns between registries. Indeed, there were no significant difference in upadacitinib and TNFi persistence in the Canadian registries (RHUMADATA: *P = *0.6601; OBRI: *P = *0.8163) but a statistically significant difference was observed in the Swiss registry (SCQM: *P = *0.0007).

### Comparison of drug persistence between upadacitinib and TNFi in patients naive and experienced to advanced treatments

**Figure 2:**
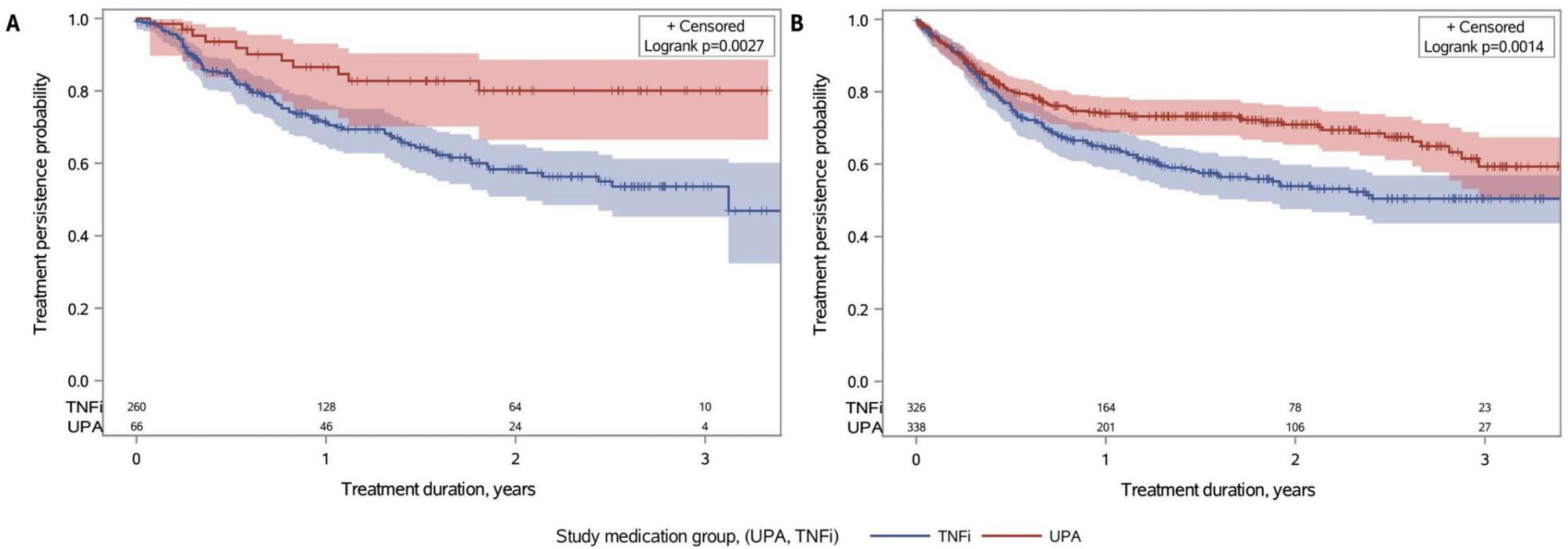
Comparison of the time to treatment discontinuation between TNFi and upadacitinib by exposure to previous treatment. (**A**) patients naive to an advanced treatment; (**B**) patients previously exposed to an advanced treatment. Advanced treatment is defined as biologics or others, excluding conventional synthetic disease-modifying antirheumatic drugs. Shaded area represents 95% CI. TNFi, tumour necrosis factor inhibitor; UPA, upadacitinib

The probability of persistence was significantly higher with upadacitinib compared with TNFi, both for patients naive to prior advanced treatments (log-rank *P = *0.0027; Fig. 2A) and for patients previously exposed to an advanced treatment (*P = *0.0014; Fig. 2B). Specifically, for patients without prior exposure to TNFi, upadacitinib showed significantly higher persistence compared with those using TNFi (*P < *0.0001, Supplementary Fig. S3A). There was no significant difference in persistence between upadacitinib and TNFi in patients previously exposed to a TNFi (*P = *0.1638; Supplementary Fig. S3B). Also, patients using upadacitinib had higher persistence than patients using TNFi when analysed by previous exposure to non-TNFi treatments including JAKi [previously exposed to non-TNFi (long-rank *P < *0.0001), previously exposed to JAKi (*P < *0.0001), naive to prior JAKi (*P = *0.0107), naive to prior non-TNFi (*P = *0.0064), Supplementary Fig. S4].

### Comparison of drug persistence between upadacitinib and TNFi when used in combination with csDMARDs at baseline

The impact of combination therapy (≥1 csDMARD) and of specific csDMARDs on persistence was assessed by adjusting the models for variables such as data source, age, gender, disease duration and number of previous advanced treatments. These analyses included 968 patients representing 97.8% of study participants.

Patients who were using a concomitant csDMARD at treatment initiation had a higher probability of persistence than patients using them as monotherapy for both upadacitinib (HR: 0.225; 95% CI: 0.107, 0.471; *P < *0.001) and TNFi (HR: 0.516; 95% CI: 0.303, 0.881; *P = *0.0152) (Table 3). In particular, the concomitant use of MTX was a significant predictor of persistence for both upadacitinib (HR: 0.604; 95% CI: 0.390, 0.934; *P = *0.0236) and TNFi (HR: 0.694; 95% CI: 0.522, 0.925; *P = *0.0126). Similarly, treatment maintenance was longer in patients using both upadacitinib and TNFi in combination with a csDMARD at baseline compared with monotherapy (Table 2). However, it is important to note that the stratification was based on the use of csDMARD solely at baseline, without collecting data on concomitant use during subsequent visits. Therefore, these results should be interpreted with caution, as they reflect csDMARD use at treatment initiation and may not represent csDMARD concomitant usage patterns during upadacitinib/TNFi treatment.

**Table 3:** Impact of combination therapy with csDMARDs on drug persistence.

	TNFi		Upadacitinib	
Variable	Hazard-ratio (95% CI)	*P* value	Hazard-ratio (95% CI)	*P* value
Concomitant use of a csDMARDs (MTX, HCQ, SSZ and LEF)	0.516 (0.303, 0.881)	0.0152	0.225 (0.107, 0.471)	<0.0001
Concomitant use of MTX (ref=monotherapy)	0.694 (0.522, 0.925)	0.0126	0.604 (0.390, 0.934)	0.0236
Concomitant use of HCQ (ref=monotherapy)	0.710 (0.419, 1.203)	0.2028	0.817 (0.450, 1.483)	0.5056
Concomitant use of SSZ (ref=monotherapy)	1.024 (0.570, 1.842)	0.9356	0.945 (0.411, 2.174)	0.8941
Concomitant use of LEF (ref=monotherapy)	1.004 (0.661, 1.526)	0.9842	1.562 (0.835, 2.919)	0.1626

All models are adjusted for data provenance, age, gender, duration of disease and number of previous advanced treatments.

cs: conventional synthetic; HCQ: hydroxychloroquine; LEF: leflunomide; MTX: methotrexate; SSZ: sulfasalazine; TNFi: tumour necrosis factor inhibitor.

### Effectiveness of upadacitinib and TNFis

The effectiveness of upadacitinib and TNFis at 6 and 12 months was observed with various outcomes measures (Table 4). Only patients with available disease activity data at baseline, 6 months and 12 months were included in this analysis. At 6 months, 70.8% of upadacitinib patients and 61.0% of TNFi patients achieved low disease activity (CDAI ≤ 10) (*P = *0.1541), with results of 70.5% and 75.2% at 12 months (*P = *0.4494). Using the HAQ-DI, the proportion of patients with normal functional ability (HAQ-DI ≤ 0.5) was similar between upadacitinib and TNFi at 6 months (42.3% *vs* 47.7%, *P = *0.4751) and at 12 months (37.5% *vs* 51.6%, *P = *0.0735). Interestingly, at 6 months, a significantly higher proportion of patients achieved minimal clinically important difference (MCID) in PtGA defined as a reduction of at least 10 mm on the PtGA score, in the TNFi group compared with the upadacitinib group (24.5% *vs* 10.7%, *P = *0.0273), but the proportions were similar at 12 months (20.5% *vs* 11.1%, *P = *0.1298). This suggests possible multidimensional aspects of response, though sample sizes were small and these findings should be interpreted cautiously. Mean effectiveness scores are presented at baseline, Months 6 and 12 in Supplementary Table S4. Although mean baseline values were generally statistically significantly higher in the upadacitinib group, comparable scores were observed at Months 6 and 12.

**Table 4:** Effectiveness of TNFi and upadacitinib at 6 and 12 months.

	6 months			12 months		
	TNFi n (%)	Upadacitinib n (%)	*P* value	TNFi n (%)	Upadacitinib n (%)	*P* value
**CDAI**, N	136	89		149	88	
Remission (≤2.8)	31 (22.8%)	25 (28.1%)	0.4309	39 (26.2%)	21 (23.9%)	0.7582
LDA (≤10)	83 (61.0%)	63 (70.8%)	0.1541	112 (75.2%)	62 (70.5%)	0.4494
MCID	9 (6.6%)	6 (6.7%)	1.0000	11 (7.4%)	6 (6.8%)	1.0000
**DAS28-CRP**, N	102	71		122	72	
≤2.6	64 (62.8%)	48 (67.6%)	0.5230	83 (68.0%)	45 (62.5%)	0.4378
≤3.2	74 (72.6%)	54 (76.1%)	0.7249	98 (80.3%)	55 (76.4%)	0.5859
**DAS28-ESR**, N	101	75		116	71	
Remission (≤2.6)	49 (48.5%)	38 (50.7%)	0.8789	61 (52.6%)	29 (40.9%)	0.1332
LDA (≤3.2)	67 (66.3%)	55 (73.3%)	0.4088	85 (73.3%)	43 (60.6%)	0.0765
**HAQ-DI**						
N (for MCID)	72	51		74	48	
MCID (≥0.22 improvement)	11 (15.3%)	6 (11.8%)	0.7916	11 (14.9%)	8 (16.7%)	0.8029
N (for NFA)	130	78		122	72	
NFA (≤0.5)	62 (47.7%)	33 (42.3%)	0.4751	63 (51.6%)	27 (37.5%)	0.0735
**Pain**, N	153	95		157	91	
MCID (≥10 mm reduction)	30 (19.6%)	13 (13.7%)	0.3006	31 (19.8%)	14 (15.4%)	0.4944
**PtGA**, N	94	75		83	72	
MCID (≥10 mm reduction)	23 (24.5%)	8 (10.7%)	0.0273	17 (20.5%)	8 (11.1%)	0.1298

MCID for CDAI was 12 for patients starting CDAI score in high disease activity (CDAI >22), six for patients starting in moderate disease activity (CDAI 10-22), and one for patients initially in low disease activity (CDAI  < 10). MCID for HAQ-DI was an improvement ≥0.22 from baseline and NFA was a score ≤0.5. MCID for pain and PtGA was a reduction ≥10 mm from baseline.

CDAI: Clinical Disease Activity Index; CRP: C-reactive protein; DAS28: Disease Activity Score—28 Joint count; ESR: erythrocyte sedimentation rate; HAQ-DI: Health Assessment Questionnaire-Disability Index; LDA: low disease activity; MCID: minimal clinically important difference; NFA: normal functional activity; PtGA: Patient Assessment of Global Disease Activity; TNFi: tumour necrosis factor inhibitor.

## Discussion

This study demonstrated that treatment persistence was longer for upadacitinib than for TNFis in a large cohort of Canadian and Swiss patients with RA. This finding was obtained using pooled data from the RHUMADATA, OBRI and SCQM registries over a 3-year period, highlighting the robustness of the dataset. Notably, upadacitinib’s longer persistence was observed despite the upadacitinib-treated cohort having more severe disease profiles compared with those on other treatments, underscoring the efficacy of upadacitinib regardless of previous exposure to other advanced treatments.

These findings are consistent with other real-world studies comparing upadacitinib and TNFi persistence. Data from the OPAL dataset, which included medical records for 7089 Australian patients with RA, revealed that upadacitinib has a longer persistence compared with other JAKis (28.8 *vs* 17.2 months, respectively) or TNFis (26.6 *vs* 13.3 months, respectively) [23]. In a previously published study using pooled data from RHUMADATA and OBRI registries, similar discontinuation rates were reported for tofacitinib and TNFis (36.7% and 37.5%, respectively) but noted that tofacitinib had reduced persistence due to adverse events compared with TNFis, with adjusted HRs for patients using TNFis of 0.46 (95% CI: 0.29–0.74; *P = *0.001) [17]. Further retrospective analysis of administrative claims databases reported that patients with RA who initiated upadacitinib were significantly more likely to adhere to their therapy and less likely to discontinue treatment within the first 12 months compared with those who initiated a TNFi (*P < *0.05) [24]. Upadacitinib demonstrated higher persistence rates than TNFi, both overall and specifically among patients with prior TNFi experience [24]. In Australian patients with RA, upadacitinib exhibited the highest persistence rates, with 12-month treatment persistence at 72% compared with 58% for TNFis [25]. A multicentre, retrospective cohort study in Japan that evaluated risk factors affecting drug persistence of JAKis in patients with RA reported that upadacitinib generally had higher drug retention rates compared with TNFi, with its persistence notably unaffected by high baseline RA disease activity; a factor that reduced persistence for TNFi. This suggests that upadacitinib may provide more consistent long-term adherence compared with TNFi, particularly for patients with more severe RA symptoms [26].

A subgroup analysis of patients previously treated with more than one mode of action before the current regimen (b/tsDMARD) also showed consistently higher persistence of upadacitinib compared with TNFi, further supporting the robustness of this finding across different clinical backgrounds.

The definition of treatment discontinuation in this study – the use of a >30-day gap for upadacitinib and >90-day gap for TNFi – was based on recommended registry methodology and real-world drug dosing frequency. Importantly, this approach likely underestimated upadacitinib persistence relative to TNFi, thereby supporting the robustness of our core finding. Still, we acknowledge that this methodology may result in minor misclassification and recommend harmonization of persistence definitions in future studies. Despite patients prescribed upadacitinib having more severe RA at baseline, using the CDAI, the effectiveness of upadacitinib and TNFis was similar at 6 and 12 months, which is consistent with a previous report that the proportions of patients with low disease activity/remission were similar for tofacitinib and TNFis after 6 months of treatment [27]. The occurrence of missing data from the disease activity indexes observed with this study is consistent with other observational studies where baseline disease activity measures were not included in many medical records [28].

A major strength of this study is the large RA population-based retrospective pooled cohort using data from distinct registries over a period of 3.5 years. The potential risk of bias in this observational study may have been mitigated through the utilization of propensity score methods, which helped achieve covariate balance between treatment arms.

However, there are important limitations of this study that must be considered. The observation of variation in persistence patterns between registries, although propensity scores were applied, underscores how unmeasured and residual confounders may persist and impact the results. Registry and country heterogeneity may be due to differences in health systems, patient characteristics, reimbursement policies and clinical practice. It would be interesting to evaluate the impact of these differences in future studies.

Data on specific adverse events leading to treatment discontinuation were not systematically available. Regulatory safety communications and non-medical reasons for withdrawal could have contributed to observed discontinuation rates, particularly for JAKi, but could not be specifically analysed. Additionally, another challenge is the presence of missing outcome data at follow-up, which was common, and as no imputation was performed for post-baseline data, this limitation – common in observational studies – can complicate data analysis and may lead to biased and inefficient estimates. Nevertheless, the results of Cox regression analyses of the complete data set remained consistent with those derived from multiple imputation (20 imputations). These analyses were adjusted for treatment attribution bias using propensity score and selected variables. However, there is still a potential for biases due to the multiple imputations and the considerable number of variables with missing data although when the analysis was repeated with 50 imputations comparable results were obtained. Also, effectiveness was evaluated using observed data only across the various disease activity indices, representing a snapshot of the available data at each time point rather than the evolution over time, while acknowledging that missing data – often due to treatment discontinuation – are part of the real‑world effectiveness profile.

Future research should include more granular data collection on the reasons for discontinuation, harmonize persistence definitions and, where possible, integrate patient-reported and objective disease activity measures to further elucidate the comparative real-world value of advanced treatments in RA.

In conclusion, patients who initiated treatment with of this study that must be considered were more likely to maintain their therapy compared with those receiving TNFi treatment. Insights gained from this real-world evidence study can inform and enhance the planning of future analyses of observational data.

## Supplementary Material

keag301_Supplementary_Data

## Data Availability

The data underlying this article were provided by RHUMADATA, OBRI and SCQM registries by permission. The SCQM informed consent foresees data sharing for research purposes. Restrictions apply to the availability of these data. Data are available after having received approval and permission from each of the registries.
