# Supplementary material for: Persistence and effectiveness of upadacitinib and TNFi for rheumatoid arthritis using pooled data from Canadian and Swiss registries
Source: Rheumatology (Oxford). 2026 Jun 23;65(6):keag301. doi: 10.1093/rheumatology/keag301 (PMC13313160; doi:10.1093/rheumatology/keag301)
Supplement: keag301_Supplementary_Data [file keag301_supplementary_data.docx]

**Supplementary material**

**Persistence and effectiveness of upadacitinib and TNFi for rheumatoid arthritis using pooled data from Canadian and Swiss registries**

Denis Choquette^1^, Axel Finckh^2,3^, Andrea Rubbert-Roth^4^, Louis Coupal^5^, Xiuying Li^6^, Sibel Zehra Aydin^7^, Helene Jonasch^8^, Roger Gaertner^9^, Dalinda Liazoghli^9^, Mohammad Movahedi^6,10^

^1^Department of Rheumatology, CHUM, University of Montréal, Montréal, QC, Canada

^2^Division of Rheumatology, Department of Medicine, Geneva University Hospital, Geneva, Switzerland

^3^Geneva Centre for Inflammation Research (GCIR), University of Geneva, Geneva, Switzerland

^4^Department of Rheumatology and Immunology, Cantonal Hospital St Gallen, St Gallen, Switzerland

^5^RHUMADATA, Institut de Recherche en Rhumatologie de Montréal, Montréal, QC, Canada

^6^ Toronto General Hospital Research Institute, University Health Network, Toronto, ON, Canada

^7^Division of Rheumatology, University of Ottawa, The Ottawa Hospital Research Institute, Ottawa, ON, Canada

^8^AbbVie AG, Cham, Zug, Switzerland

^9^AbbVie Corporation, Saint-Laurent, QC, Canada

^10^Institute of Health Policy, Management and Evaluation, University of Toronto, Toronto, ON, Canada

**Corresponding author details:**

Full name: Denis Choquette

Postal address: Department of Rheumatology (CHUM)

1000 Saint-Denis St, Montréal, QC, H2X 3J4, Canada

Email address: [denischoquette1@gmail.com](mailto:denischoquette1@gmail.com)

**Supplementary Table S1. Data Registry Information**

| **Registry** | **Description** |
| --- | --- |
| RHUMADATA^a^ | The RHUMADATA clinical database and registry monitors the clinical care of all the patients with inflammatory diseases seen at the Institut de Rhumatologie de Montréal (IRM), the Centre de l’ostéoporose et de rhumatologie de Québec (CORQ), and the Clinique de santé Jacques-Cartier (CSJC), the largest rheumatological clinics in the province of Québec, Canada. RHUMADATA has been collecting real-world observational data since 1998. The database currently includes the treatment history of more than 6000 patients with inflammatory disease (rheumatoid arthritis [RA], ankylosing spondylitis, and spondyloarthritis). Data collected at all visits include demographics, disease history, laboratory values (rheumatoid factor [RF], anti-citrullinated peptide antibody [ACPA], C-reactive protein [CRP] and erythrocyte sedimentation rate [ESR]), all disease activity scores (disease activity score [DAS] – CRP and ESR, Clinical Disease Activity Index [CDAI] and simplified disease activity index [SDAI]), patient report outcomes (PROs) including health assessment questionnaire-disability index (HAQ-DI), morning stiffness, patient global evaluation of disease activity, patient evaluation of pain, and physician global evaluation of disease activity. Comorbidities including, but not limited to, cardiovascular disease, diabetes, high blood pressure, cancer, and infections are also collected. Medication usage for disease control is entered in the database (start and termination data and a reason for termination). The RHUMADATA protocol was approved by the Advarra IRB. |
| OBRI^b^ | The Ontario Best Practices Research Initiative (OBRI) is a multicentre registry across Ontario, Canada, collecting data from rheumatologists and patients with RA at enrolment and follow-up. It incorporates rheumatologist assessments from approximately one-third of the rheumatologists in Ontario. Patients are eligible to be enrolled if they are ≥16 years of age at the time of diagnosis, ≥18 years of age at enrolment, have a rheumatologist-confirmed RA diagnosis and have at least one swollen joint. Enrolled patients are interviewed every 6 months by phone and seen by their rheumatologist in routine care. Patients are asked for their general medical history and comorbidity status at enrolment. Rheumatologists are also expected to report any history of previous comorbidity, including all disease and RA disease activity such as inflammatory markers, patient and physician global assessment, and tender and swollen joint counts. Data on socio-demographics, smoking status, height, weight, and any prior and current medications are recorded during the rheumatologist enrolment visit or the patient’s interview. Patient-reported outcomes for functional status are also collected. At follow-up visits, all the information mentioned previously is updated. RA medication changes (including discontinuation and reasons for discontinuation) between visits are also captured. Rheumatologists report any incident of comorbidity and reassess disease activity during every follow-up visit. All sites in the OBRI had ethics approval to enrol patients. |
| SCQM^c^ | The Swiss Clinical Quality Management in Rheumatic Diseases (SCQM) cohort can be considered a population-based sample in terms of targeted antirheumatic therapies. The clinical information is collected by the treating rheumatologist and the patient. This information includes patient demographics, lifestyle habits, clinical endpoints such as disease activity, safety outcomes, medication use, and patient-reported outcomes. A national review board (Geneva Ethical Committee) has approved the SCQM registry, and all participants gave informed consent before enrolment in accordance with the Declaration of Helsinki.  The informed consent that patients sign upon being included in SCQM includes a statement on who is authorized to use the data: “Your encrypted/pseudonymized or anonymized data and/or your encrypted/pseudonymized blood samples will be made available to authorized researchers for research projects or may be used in research projects in collaboration with other public or private institutions (other hospitals, universities or pharmaceutical companies). The projects can be carried out in Switzerland or abroad and may include genetic analyses. For research projects abroad, at least the same data protection requirements apply as in Switzerland. Research projects are generally subject to review by the responsible ethics committee.” |

^a^RHUMADATA. RHUMADATA Inflammatory disease registry, Montreal, QC, Canada. ^b^OBRI. Ontario Best Practices Research Initiative [Available from: <http://www.obri.ca>]. ^c^SCQM. Swiss Clinical Quality Management in Rheumatic Diseases registry [Available from: <https://www.scqm.ch/en/>].

**Supplementary Table S2. List of Variables that were Imputed with Proportion of Available Data from the Combined RHUMADATA, OBRI, and SCQM Databases**

| **Variable** | **Treatment initiation**  **(% of data that were available)** |
| --- | --- |
| **N** | **990** |
| Patient number | 100.0% |
| Gender (man, woman) | 100.0% |
| Age (at treatment initiation), years | 100.0% |
| Study medication (upadacitinib, tumour necrosis factor inhibitor) | 100.0% |
| TNFi used (adalimumab, certolizumab pegol, etanercept, golimumab, infliximab) | 100.0% |
| Reference molecule or biosimilar | 100.0% |
| Start date of study medication | 100.0% |
| Years of education, years | 82.2% |
| Smoking status (non-smoker, ever smoked [i.e., smoker or ex-smoker]) | 84.7% |
| Disease duration (from diagnosis to treatment initiation in years) | 97.8% |
| Rheumatoid Factor | 54.1% |
| Anti Citrullinated Peptide | 41.2% |
| Erythrocyte Sedimentation Rate (mm/hr) | 53.8% |
| C-reactive protein (mg/L) | 65.3% |
| Patient Global Assessment of rheumatoid arthritis activity, 0 to 10 visual analog scale (PtGA) | 47.1% |
| Physician Global Assessment of rheumatoid arthritis activity, 0 to 10 visual analog scale (PGA) | 53.0% |
| Swollen Joint Count-28, 0 to 28 swollen joints | 55.8% |
| Tender Joint Count-28, 0 to 28 tender joints | 54.8% |
| Clinical Disease Activity Index (CDAI) | 40.9% |
| Disease Activity Score-28 Erythrocyte Sedimentation Rate (DAS(4)ESR) | 29.8% |
| Health Assessment Questionnaire – Disability Index (HAQ-DI) | 38.7% |
| Patient-reported pain, 0 to 10 visual analog scale | 47.7% |
| Patient-reported fatigue, 0 to 10 visual analog scale | 17.0% |
| Hypertension (present or absent at treatment initiation) | 87.1% |
| Cardiovascular disease (present or absent at treatment initiation) | 79.5% |
| Diabetes Mellitus (present or absent at treatment initiation) | 80.4% |
| Lung diseases (present or absent at treatment initiation) | 83.0% |
| Gastrointestinal disease (present or absent at treatment initiation) | 85.8% |
| Cancer disease (present or absent at treatment initiation) | 90.9% |
| Depression (present or absent at treatment initiation) | 87.4% |
| Prior use of a tumour necrosis factor inhibitor | 100.0% |
| Prior use of a non- tumour necrosis factor inhibitor | 100.0% |
| Prior use of a Janus kinase inhibitor | 100.0% |
| Previous number of advanced treatments (biologics or others excluding csDMARDs) | 100.0% |
| Concomitant use of hydroxychloroquine | 99.9% |
| Concomitant use of leflunomide | 100.0% |
| Concomitant use of methotrexate | 99.9% |
| Concomitant use of sulfasalazine | 100.0% |
| Concomitant use of nonsteroidal anti-inflammatory agents | 54.9% |
| Concomitant use of oral steroids | 100.0% |

**Supplementary Table S3. Multivariable Cox Regression Analysis for Treatment Discontinuation**

| **Variables** |  | **Hazard-ratio  and 95% CI** | ***P* value** |
| --- | --- | --- | --- |
| **Cox Regression Performed on Multiple Imputation Data (20 datasets)** | | | |
| Study medication group (reference=TNFi) | UPA | 0.602 (0.469, 0.773) | <0.0001 |
| Data provenance (reference=RHUMADATA) | OBRI | 0.476 (0.311, 0.728) | 0.0006 |
|  | SCQM | 1.660 (1.187, 2.321) | 0.0031 |
| Age at treatment initiation, years |  | 0.997 (0.988, 1.006) | 0.5117 |
| Gender (reference=Man) | Woman | 1.218 (0.913, 1.625) | 0.1801 |
| Smoking status (reference=Never smoker) | Ever | 1.017 (0.790, 1.310) | 0.8942 |
| Years of education, years |  | 1.007 (0.947, 1.072) | 0.8208 |
| Seropositivity (reference=RF- and ACPA-) | Positive | 0.849 (0.643, 1.120) | 0.2458 |
| Combination therapy (reference=Monotherapy) | Combo therapy | 0.730 (0.585, 0.912) | 0.0055 |
| Disease duration, years |  | 0.980 (0.967, 0.994) | 0.0041 |
| Number of previous advanced treatments |  | 1.111 (1.048, 1.177) | 0.0004 |
| HAQ-DI |  | 1.224 (0.988, 1.517) | 0.0648 |
| **Cox Regression Performed on Multiple Imputation Data Adjusted for Treatment Attribution Bias (Propensity Score) Model 1. Adjusting the primary outcome for propensity score alone** | | | |
| PS score |  | 1.340 (0.775, 2.316) | 0.2950 |
| Study medication group (reference=TNFi) | UPA | 0.606 (0.469, 0.783) | 0.0001 |
| **Model 2. Adjusting the primary outcome for PS score and selected variables** | | | |
| PS score |  | 2.604 (0.825, 8.223) | 0.1026 |
| Study medication group (reference=TNFi) | UPA | 0.568 (0.439, 0.736) | <0.0001 |
| Data provenance (reference=RHUMADATA) | OBRI | 0.525 (0.337, 0.817) | 0.0043 |
|  | SCQM | 1.958 (1.326, 2.892) | 0.0007 |
| Age at treatment initiation, years |  | 0.994 (0.985, 1.004) | 0.2241 |
| Gender (reference=Man) | Woman | 1.224 (0.918, 1.632) | 0.1689 |
| Smoking status (reference=Never smoker) | Ever | 1.012 (0.785, 1.304) | 0.9278 |
| Years of education, years |  | 1.010 (0.947, 1.077) | 0.7554 |
| Seropositivity (reference=RF- and ACPA-) | Positive | 0.812 (0.615, 1.073) | 0.1426 |
| Combination therapy (reference=Monotherapy) | Combo therapy | 0.744 (0.596, 0.930) | 0.0095 |
| Disease duration, years |  | 0.979 (0.966, 0.993) | 0.0024 |
| Number of previous advanced treatments |  | 1.034 (0.932, 1.147) | 0.5314 |
| HAQ-DI |  | 1.220 (0.982, 1.515) | 0.0726 |
| The propensity scores were obtained using the following baseline variables: age, disease duration, number of previous advanced treatments, data provenance, seropositivity (RF+ or ACPA+), history of hypertension, diabetes mellitus, anxiety/depression, lung disease, prior use of TNFi, prior use of a non-TNFi, prior use of JAKi, concomitant use of methotrexate, concomitant use of hydroxychloroquine, concomitant use of oral steroids, ESR, CRP, patient-reported pain, patient global assessment of disease activity, physician global assessment of disease activity, tender and swollen joint counts, DAS28(4)ESR, HAQ-DI, and CDAI.  ACPA+/-, anti-citrullinated peptide positive/negative; CDAI, Clinical Disease Activity Index; CRP, C-reactive protein; DAS28, Disease Activity Score – 28 Joint count; ESR, erythrocyte sedimentation rate; HAQ-DI, Health Assessment Questionnaire-Disability Index; JAKi, Janus kinase inhibitor; OBRI, Ontario Best Practices Research Initiative; RF+/-, rheumatoid factor positive/negative; SCQM, Swiss Clinical Quality Management in Rheumatic Diseases; TNFi, tumour necrosis factor inhibitor; UPA, upadacitinib. | | | |

**Supplementary Table S4. Cox Regression Models Derived from Non-missing Data Adjusting for Selected Variables**

| **Variables** |  | **Hazard-ratio**  **and 95% CI** | ***P* value** |
| --- | --- | --- | --- |
| **Model 1, N=237 (24% of data).** | | | |
| Study medication group (reference=TNFi) | UPA | 0.520 (0.300, 0.904) | 0.0203 |
| Data provenance (reference=RHUMADATA) | OBRI | 0.696 (0.339, 1.427) | 0.3223 |
|  | SCQM | 2.028 (1.085, 3.792) | 0.0267 |
| Age at treatment initiation, years |  | 0.996 (0.977, 1.015) | 0.6526 |
| Gender (reference=Man) | Woman | 1.506 (0.781, 2.904) | 0.2219 |
| Smoking status (reference=Never smoker) | Ever | 1.000 (0.595, 1.680) | 0.9997 |
| Years of education, years |  | 1.010 (0.908, 1.124) | 0.8544 |
| Seropositivity (reference=RF- and ACPA-) | Positive | 0.693 (0.425, 1.131) | 0.1420 |
| Combination therapy (reference=Monotherapy) | Combo therapy | 0.825 (0.497, 1.370) | 0.4579 |
| Disease duration, years |  | 0.968 (0.934, 1.003) | 0.0688 |
| Number of previous advanced treatments |  | 1.030 (0.880, 1.205) | 0.7102 |
| HAQ-DI |  | 1.463 (0.969, 2.208) | 0.0703 |
| **Model 2, Smoking status, years of education, and seropositivity removed from Model 1. N=373 (38% of data).** | | | |
| Study medication group (reference=TNFi) | UPA | 0.597 (0.395, 0.901) | 0.0139 |
| Data provenance (reference=RHUMADATA) | OBRI | 0.649 (0.334, 1.262) | 0.2027 |
|  | SCQM | 2.161 (1.364, 3.424) | 0.0010 |
| Age at treatment initiation, years |  | 0.991 (0.977, 1.004) | 0.1650 |
| Gender (reference=Man) | Woman | 0.939 (0.612, 1.440) | 0.7727 |
| Combination therapy (reference=Monotherapy) | Combo therapy | 0.735 (0.518, 1.043) | 0.0849 |
| Disease duration, years |  | 0.975 (0.952, 0.998) | 0.0303 |
| Number of previous advanced treatments |  | 1.105 (0.997, 1.225) | 0.0569 |
| HAQ-DI |  | 1.432 (1.059, 1.937) | 0.0196 |
| **Model 3, HAQ-DI removed from Model 2. N=968 (98% of data).** | | | |
| Study medication group (reference=TNFi) | UPA | 0.598 (0.465, 0.769) | <0.0001 |
| Data provenance (reference=RHUMADATA) | OBRI | 0.455 (0.301, 0.689) | 0.0002 |
|  | SCQM | 1.418 (1.051, 1.912) | 0.0222 |
| Age at treatment initiation, years |  | 0.999 (0.991, 1.008) | 0.8831 |
| Gender (reference=Man) | Woman | 1.227 (0.928, 1.622) | 0.1506 |
| Combination therapy (reference=Monotherapy) | Combo therapy | 0.728 (0.583, 0.910) | 0.0054 |
| Disease duration, years |  | 0.978 (0.965, 0.992) | 0.0017 |
| Number of previous advanced treatments |  | 1.137 (1.077, 1.202) | <0.0001 |
| ACPA-, anti-citrullinated peptide negative; HAQ-DI, Health Assessment Questionnaire-Disability Index; OBRI, Ontario Best Practices Research Initiative; RF-, rheumatoid factor negative; SCQM, Swiss Clinical Quality Management in Rheumatic Diseases; TNFi, tumour necrosis factor inhibitor; UPA, upadacitinib. | | | |

**Supplementary Table S5. Selected Variables at Baseline, 6, and 12 Months**

|  | **TNFi (N=586)** | **UPADACITINIB (N=404)** | ***P* value ^b^** | **Total (N=990)** |
| --- | --- | --- | --- | --- |
| **Baseline** | | | | |
| ESR (mm/h) ^a^ | 20.70 ± 18.22 (N=318) | 23.76 ± 21.42 (N=215) | 0.15 | 21.93 ± 19.61 (N=533) |
| CRP (mg/L) ^a^ | 7.19 ± 10.45 (N=385) | 9.45 ± 18.01 (N=261) | 0.12 | 8.10 ± 14.04 (N=646) |
| Patient-reported pain ^a^ | 4.33 ± 2.88 (N=287) | 4.97 ± 2.65 (N=185) | 0.026 | 4.58 ± 2.81 (N=472) |
| PtGA ^a^ | 4.48 ± 2.58 (N=263) | 5.13 ± 2.24 (N=203) | 0.0074 | 4.76 ± 2.46 (N=466) |
| PGA ^a^ | 3.92 ± 2.34 (N=323) | 4.89 ± 2.23 (N=202) | <0.0001 | 4.29 ± 2.34 (N=525) |
| TJC28 ^a^ | 4.51 ± 5.17 (N=342) | 6.21 ± 5.87 (N=201) | 0.0003 | 5.14 ± 5.50 (N=543) |
| SJC28 ^a^ | 4.26 ± 5.03 (N=347) | 5.57 ± 4.58 (N=205) | <0.0001 | 4.75 ± 4.91 (N=552) |
| DAS28(3) ESR | 3.61 ± 1.32 (N=258) | 4.25 ± 1.30 (N=157) | <.0001 | 3.86 ± 1.35 (N=415) |
| DAS28(4) ESR ^a^ | 3.81 ± 1.46 (N=241) | 4.54 ± 1.31 (N=147) | <.0001 | 4.09 ± 1.45 (N=388) |
| HAQ-DI ^a^ | 0.87 ± 0.67 (N=232) | 1.09 ± 0.71 (N=151) | 0.0031 | 0.96 ± 0.69 (N=383) |
| CDAI ^a^ | 17.27 ± 12.17 (N=260) | 20.99 ± 11.05 (N=145) | 0.0007 | 18.60 ± 11.90 (N=405) |
| **Month 6** | | | | |
| ESR (mm/h) | 20.29 ± 17.09 (N=172) | 17.58 ± 16.03 (N=118) | 0.66 | 17.54 ± 16.64 (N=290) |
| CRP(mg/L) | 5.31 ± 8.61 (N=204) | 5.09 ± 10.21 (N=152) | 0.14 | 5.22 ± 9.32 (N=356) |
| Patient-reported pain | 2.96 ± 2.64 (N=203) | 3.34 ± 2.63 (N=119) | 0.14 | 3.10 ± 2.64 (N=322) |
| PtGA | 3.38 ± 2.58 (N=154) | 3.17 ± 2.67 (N=115) | 0.44 | 3.29 ± 2.61 (N=269) |
| PGA | 2.40 ± 1.88 (N=164) | 2.17 ± 2.02 (N=112) | 0.19 | 2.30 ± 1.94 (N=276) |
| TJC28 | 2.41 ± 3.60 (N=170) | 2.63 ± 4.44 (N=116) | 0.77 | 2.50 ± 3.96 (N=286) |
| SJC28 | 1.61 ± 2.87 (N=172) | 1.59 ± 2.79 (N=118) | 0.79 | 1.60 ± 2.83 (N=290) |
| DAS28(3) ESR | 2.81 ± 1.31 (N=139) | 2.90 ± 1.23 (N=85) | 0.66 | 2.85 ± 1.27 (N=224) |
| DAS28(4) ESR | 2.86 ± 1.42 (N=120) | 2.84 ± 1.28 (N=79) | 0.95 | 2.86 ± 1.36 (N=199) |
| HAQ-DI | 0.71 ± 0.65 (N=130) | 0.91 ± 0.75 (N=78) | 0.07 | 0.79 ± 0.69 (N=208) |
| CDAI | 9.56 ± 8.58 (N=136) | 9.00 ± 10.62 (N=89) | 0.23 | 9.34 ± 9.42 (N=225) |
| **Month 12** | | | | |
| ESR (mm/h) | 15.93 ± 15.30 (N=156) | 16.86 ± 15.71 (N=94) | 0.42 | 16.28 ± 15.43 (N=250) |
| CRP (mg/L) | 4.65 ± 9.95 (N=190) | 3.93 ± 6.95 (N=122) | 0.21 | 4.37 ± 8.89 (N=312) |
| Patient-reported pain | 2.75 ± 2.50 (N=202) | 3.22 ± 2.46 (N=109) | 0.073 | 2.92 ± 2.50 (N=311) |
| PtGA | 2.84 ± 2.18 (N=169) | 3.05 ± 2.37 (N=110) | 0.57 | 2.92 ± 2.25 (N=279) |
| PGA | 1.90 ± 1.64 (N=169) | 2.08 ± 1.81 (N=102) | 0.53 | 1.97 ± 1.70 (N=271) |
| TJC28 | 1.72 ± 2.95 (N=170) | 2.35 ± 3.97 (N=100) | 0.42 | 1.96 ± 3.37 (N=270) |
| SJC28 | 1.31 ± 2.54 (N=174) | 1.89 ± 3.46 (N=100) | 0.20 | 1.52 ± 2.92 (N=274) |
| DAS28(3) ESR | 2.61 ± 1.24 (N=133) | 2.85 ± 1.30 (N=70) | 0.21 | 2.69 ± 1.26 (N=203) |
| DAS28(4) ESR | 2.63 ± 1.26 (N=120) | 3.05 ± 1.40 (N=70) | 0.056 | 2.78 ± 1.33 (N=190) |
| CDAI | 0.62 ± 0.59 (N=122) | 0.92 ± 0.68 (N=72) | 0.003 | 0.74 ± 0.64 (N=194) |
| HAQ-DI | 7.60 ± 7.02 (N=149) | 9.01 ± 8.65 (N=88) | 0.26 | 8.12 ± 7.67 (N=237) |
| Results are presented as mean ± standard deviation (n). ^a^ Covariates used to derive propensity scores. ^b^ *P* values are based on the Mann-Whitney test.  CDAI, Crohn’s Disease Activity Index; CRP, C-reactive protein; DAS28, Disease Activity Score – 28 Joint count; ESR, erythrocyte sedimentation rate; HAQ-DI, Health assessment questionnaire – disease index; PGA, Physician Global Assessment of Disease Activity; PtGA, Patient Global Assessment of Disease Activity; SJC28, 28-swollen joint count; TJC28, 28-tender joint count; TNFi, tumour necrosis factor inhibitor. | | | | |

**Supplementary Figures**


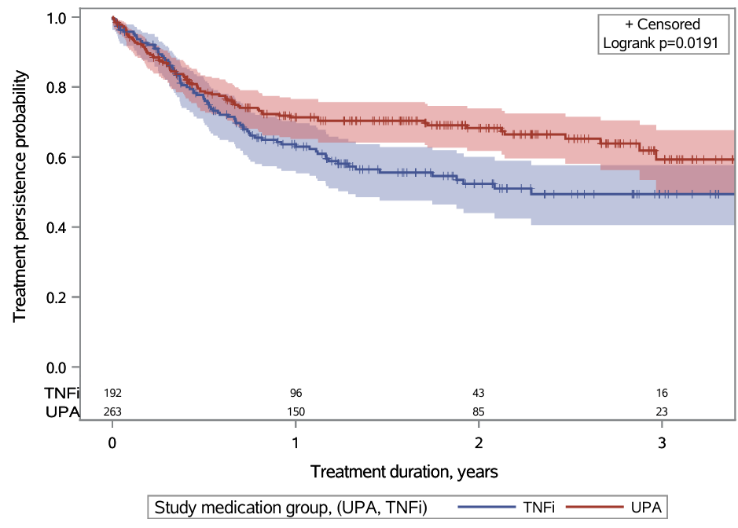

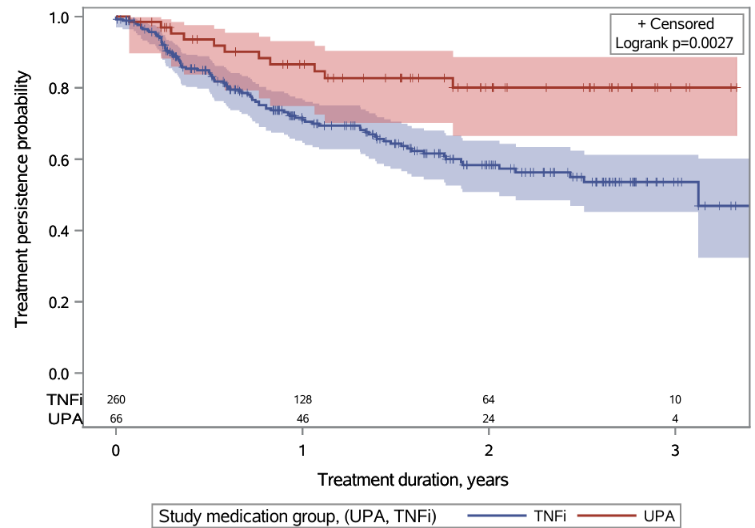

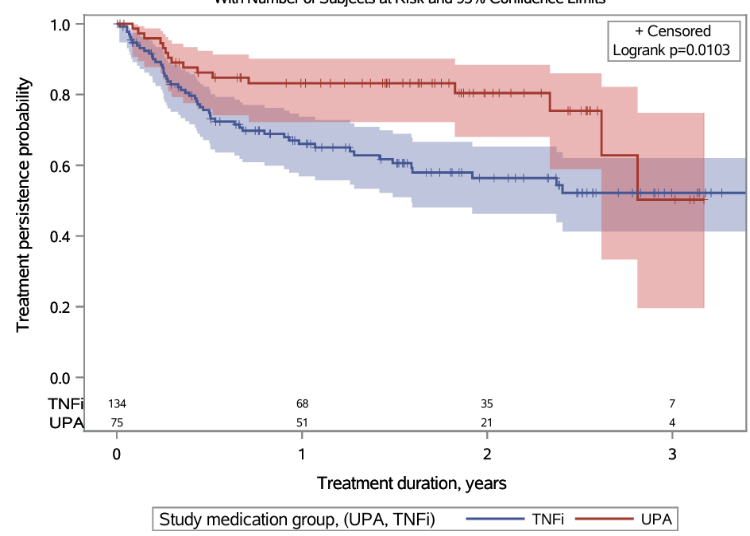


**B**

**A**


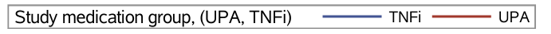


**C**


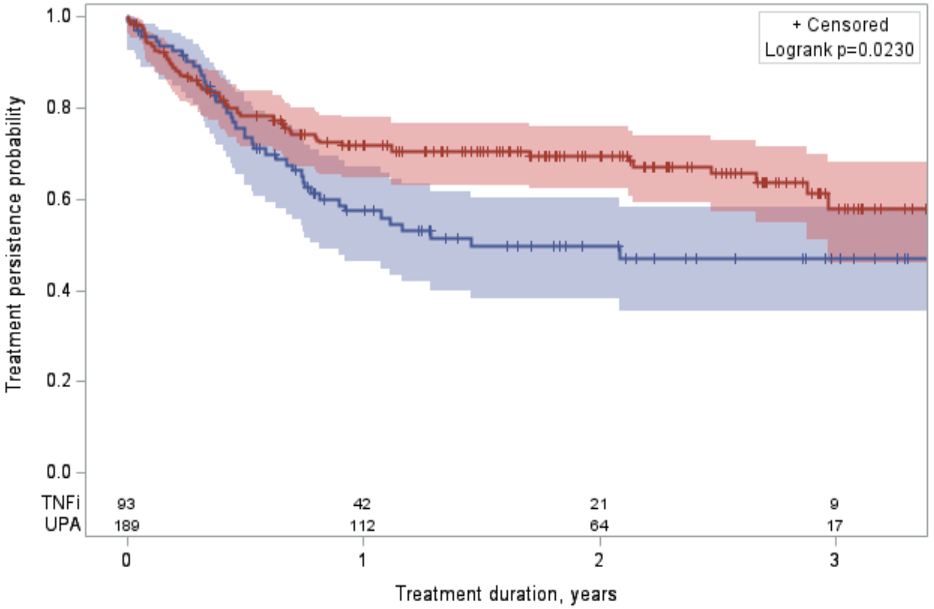


**D**

**Supplementary Figure S1. Comparison of time to treatment discontinuation between TNFi and upadacitinib by line of advanced treatment**. Patients for whom it was the (**A**) first line of advanced treatment, (**B**) second line of advanced treatment, **(C)** the third line or more of advanced treatment, and **(D)** in patients who had used more than 1 mode of action. Shaded area represents 95% CI. TNFi, tumour necrosis factor inhibitor; UPA, upadacitinib


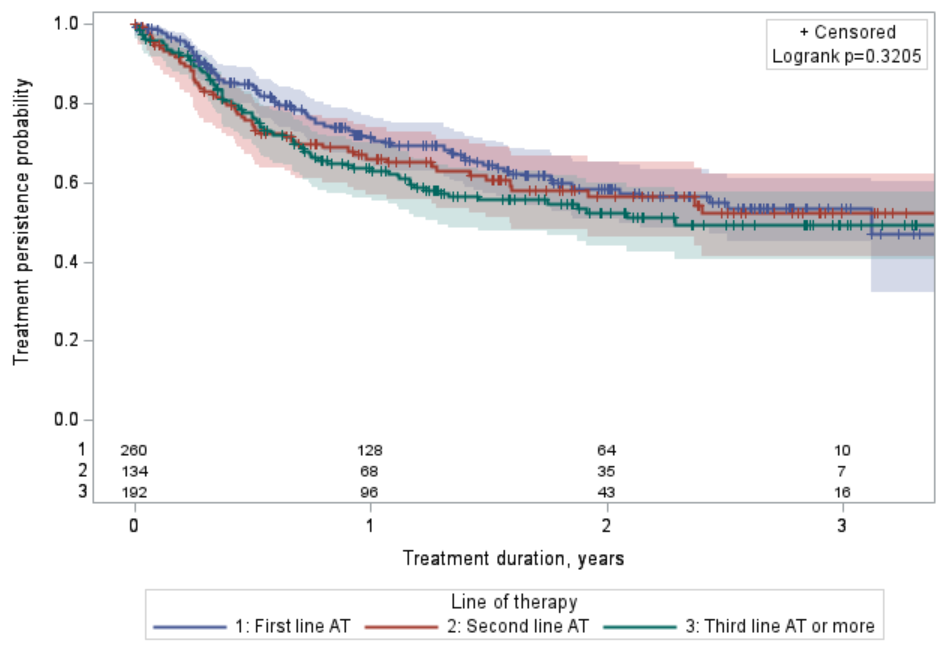


**B**


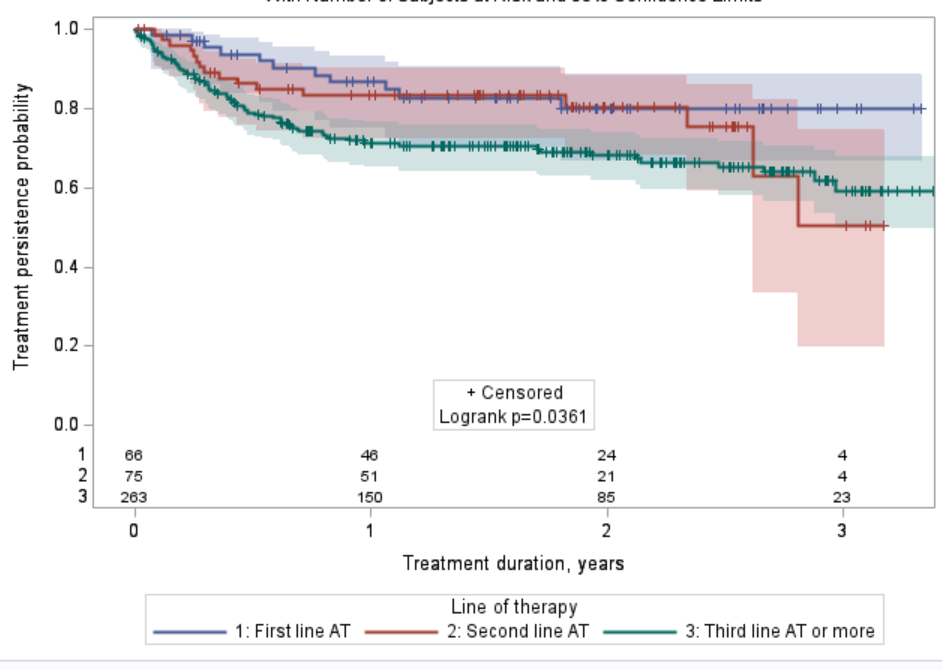


**A**


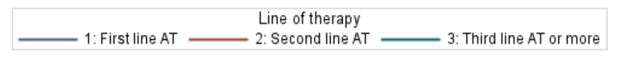


| **Adjustment for Multiple Comparisons for Log-rank Test for TNFi** | |  | **Adjustment for Multiple Comparisons for Log-rank Test for UPA** | |
| --- | --- | --- | --- | --- |
| **Strata comparison** | **P value (Sidak)** |  | **Strata comparison** | **P value (Sidak)** |
| First line AT vs second line | 0.6775 |  | First line AT vs second line | 0.9121 |
| First line AT vs third line or more | 0.3469 |  | First line AT vs third line or more | 0.0319 |
| Second line AT vs third line or more | 0.8643 |  | Second line AT vs third line or more | 0.1149 |

**Supplementary Figure S2. Comparison of time to treatment discontinuation between line of advanced treatment for TNFi and Upadacitinib.** (**A**) TNFi, (**B**) upadacitinib. Shaded area represents 95% CI. AT, advanced treatment; TNFi, tumour necrosis factor inhibitor; UPA, upadacitinib.


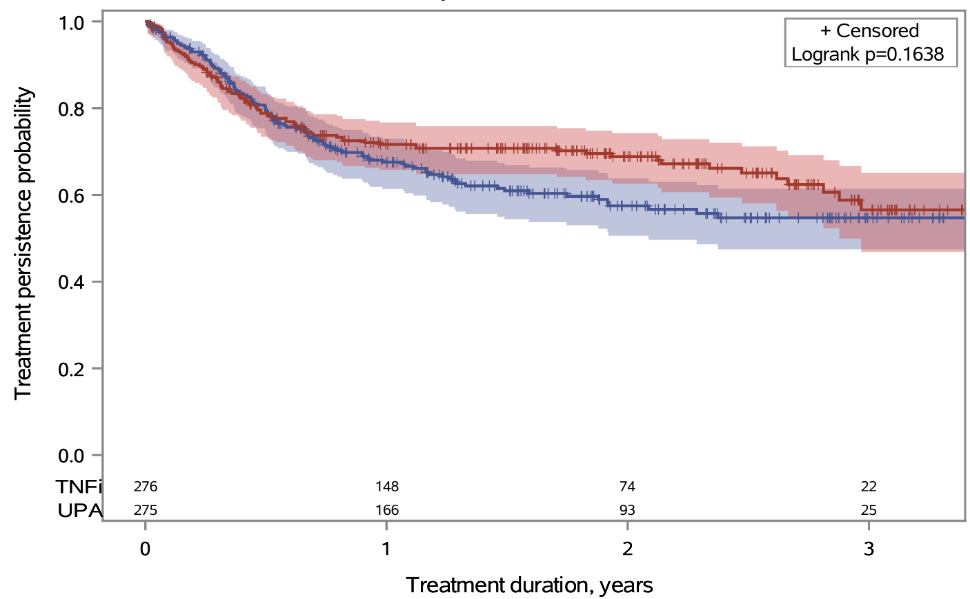

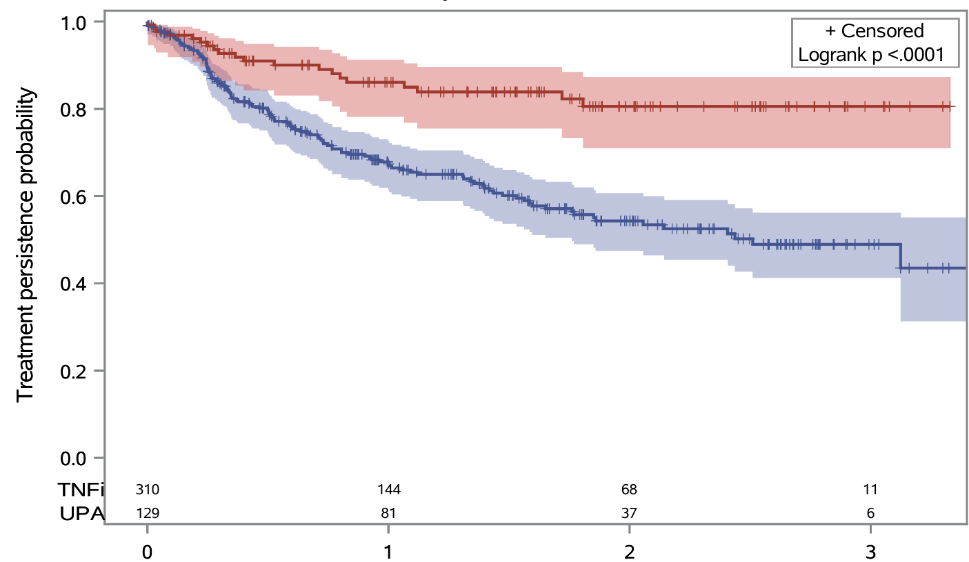


**A**

**B**


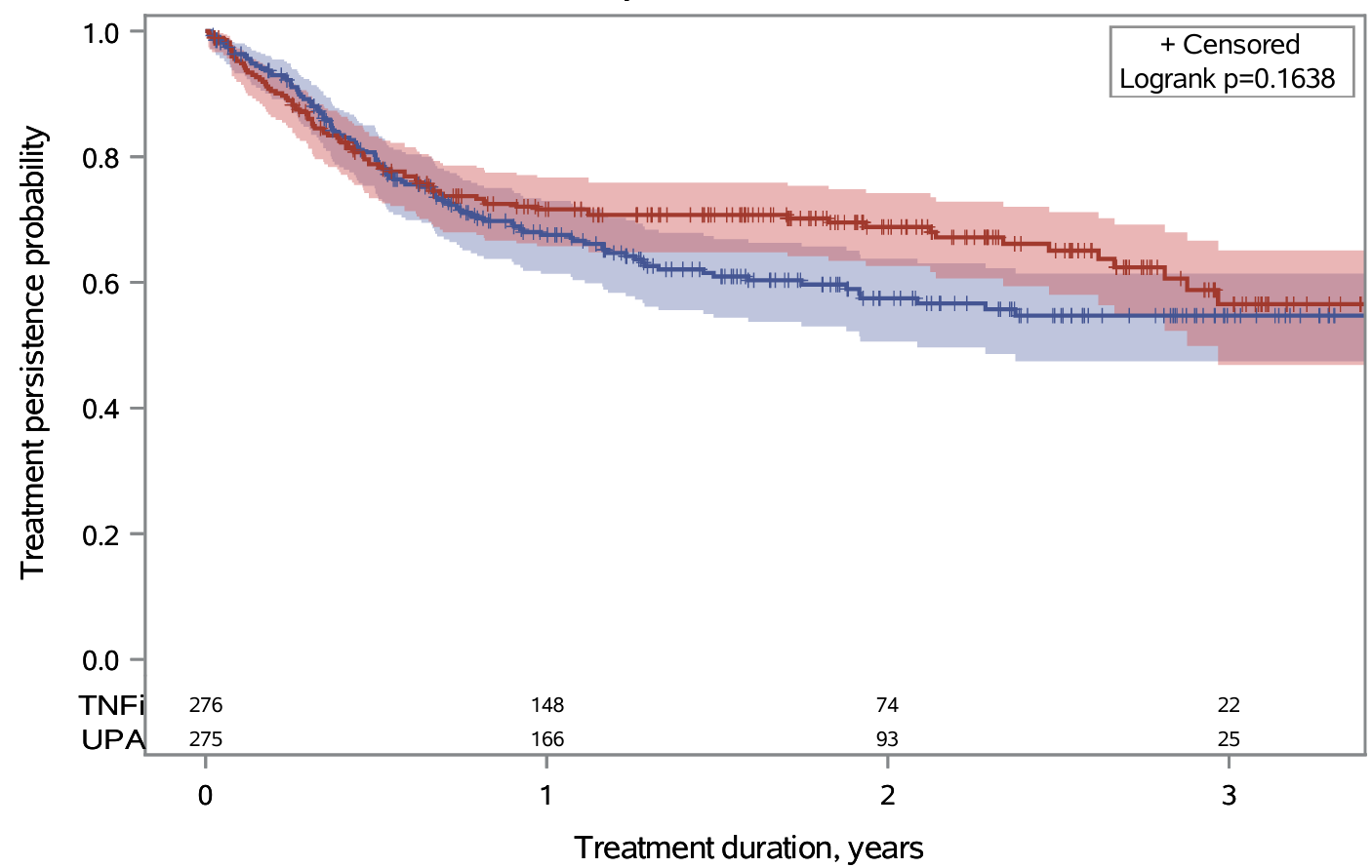

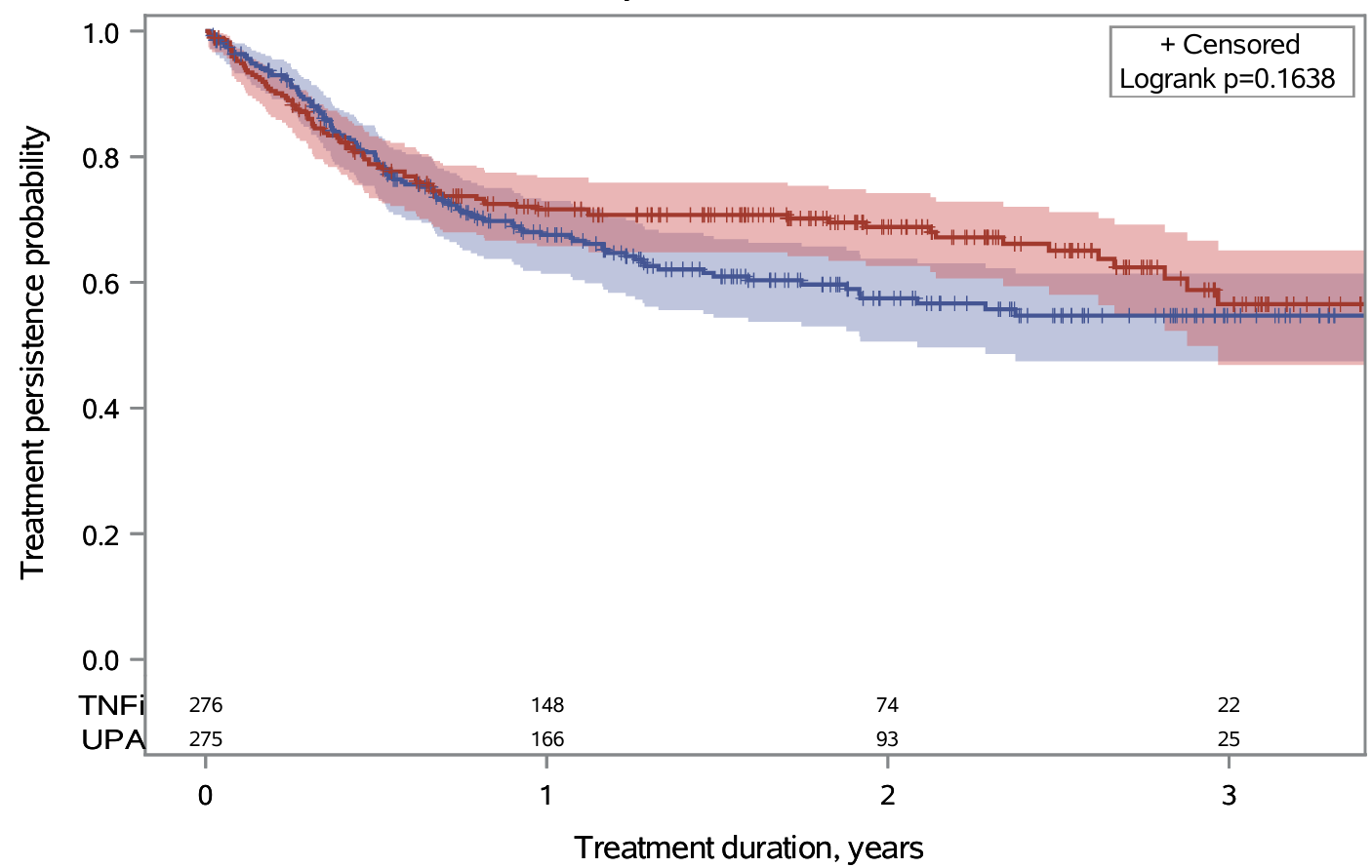

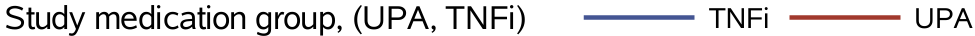


**Supplementary Figure S3. Comparison of the time to treatment discontinuation between TNFi and upadacitinib by previous exposure to TNFi**. (**A**) Patients naïve to prior TNFi (first treatment or no previous TNFi); (**B**) patients previously exposed to a TNFi. Shaded area represents 95% CI. TNFi, tumour necrosis factor inhibitor; UPA, upadacitinib.


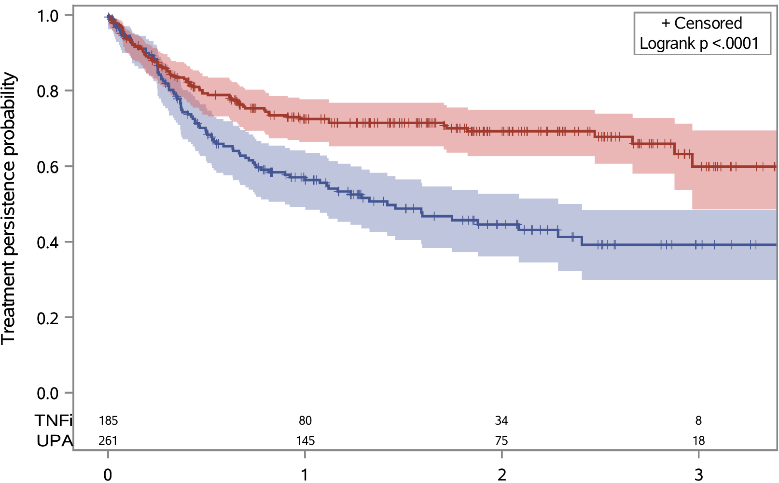

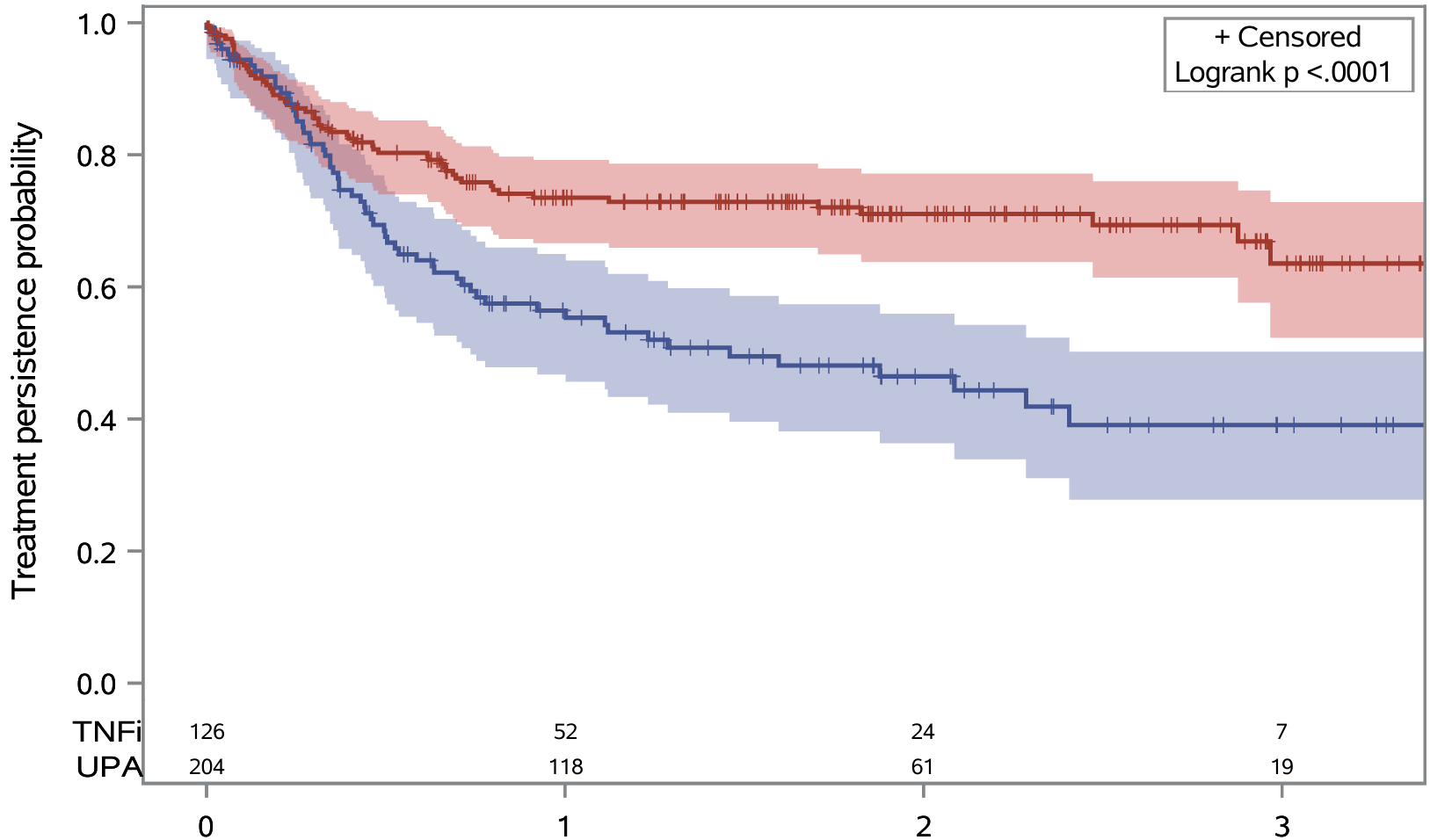

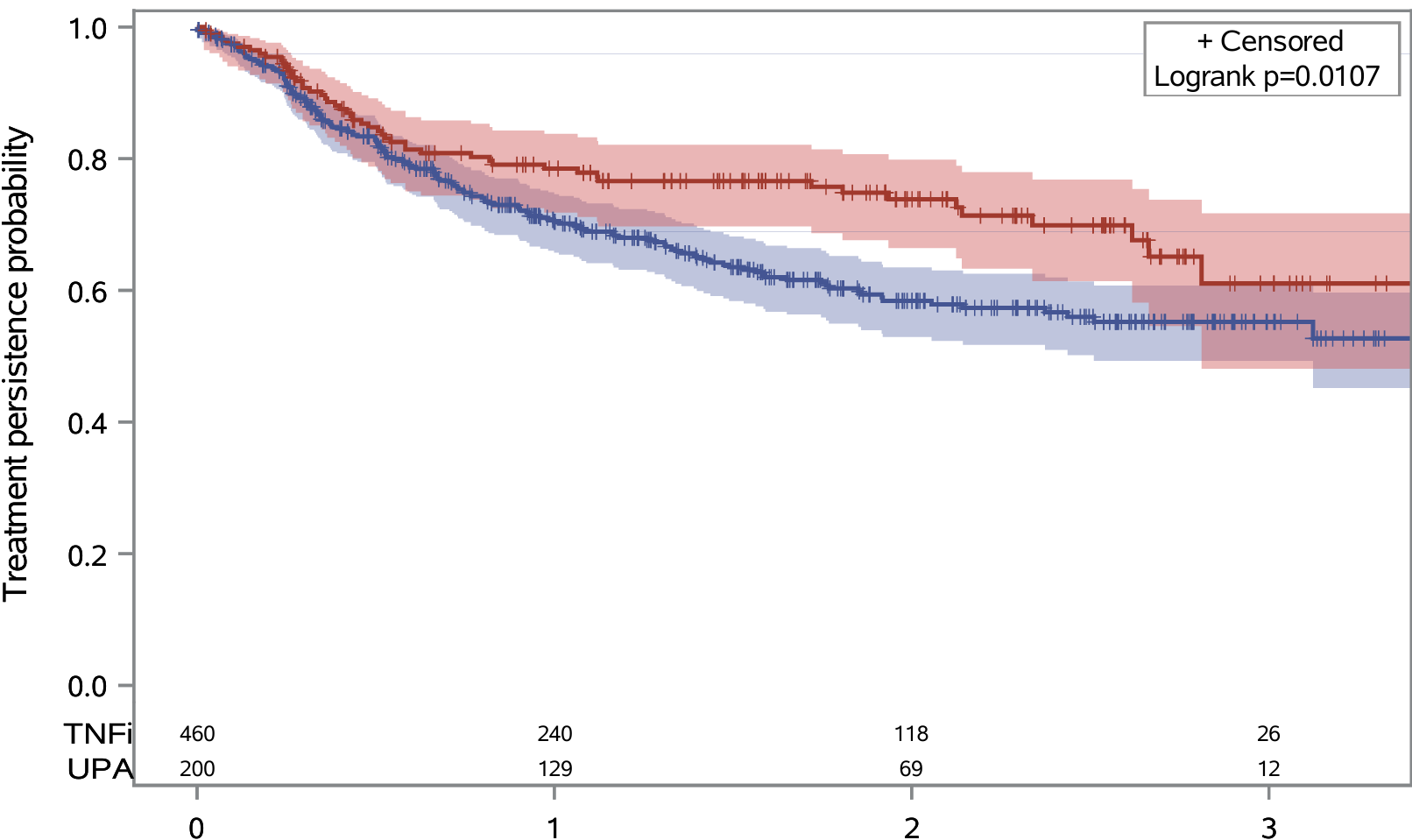

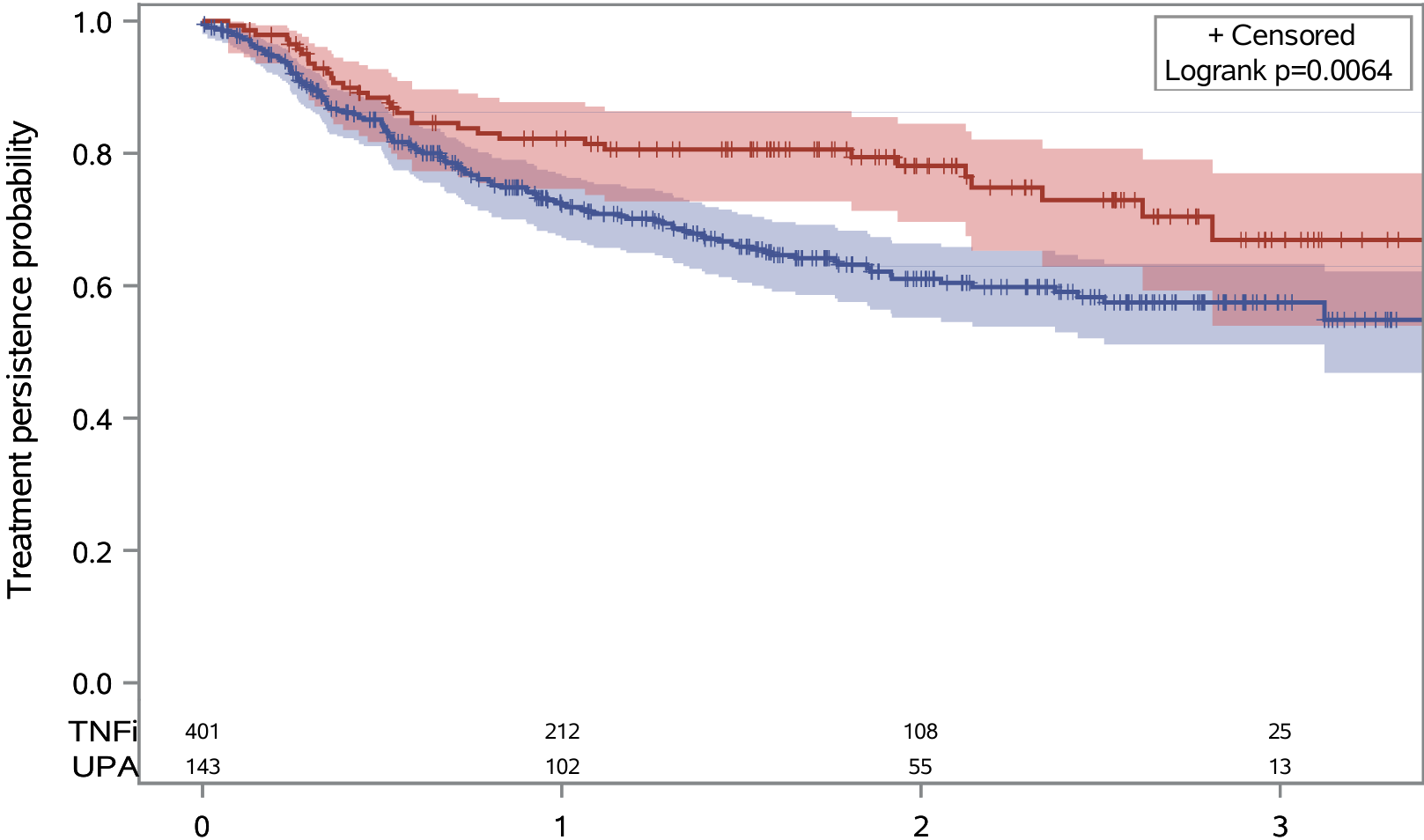

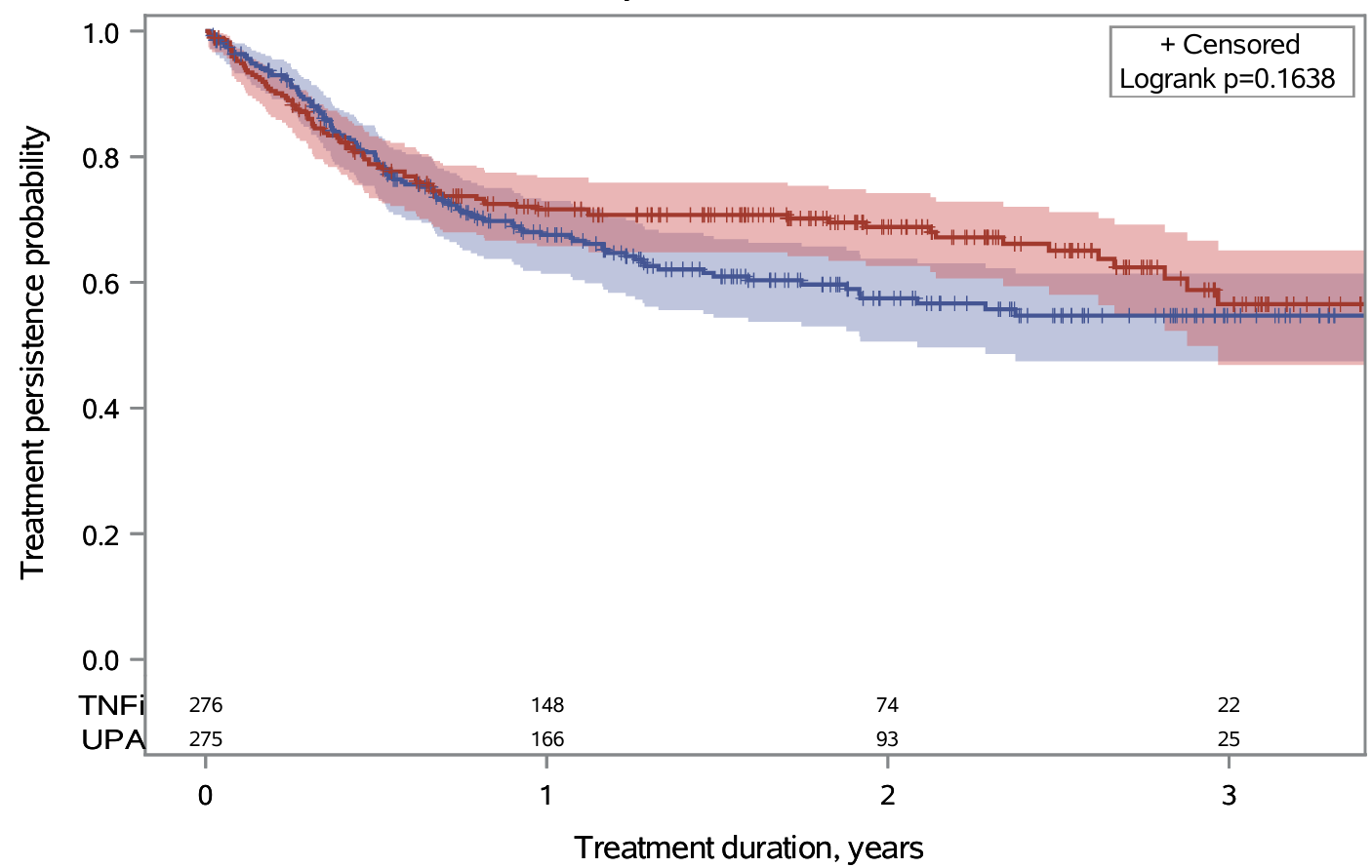

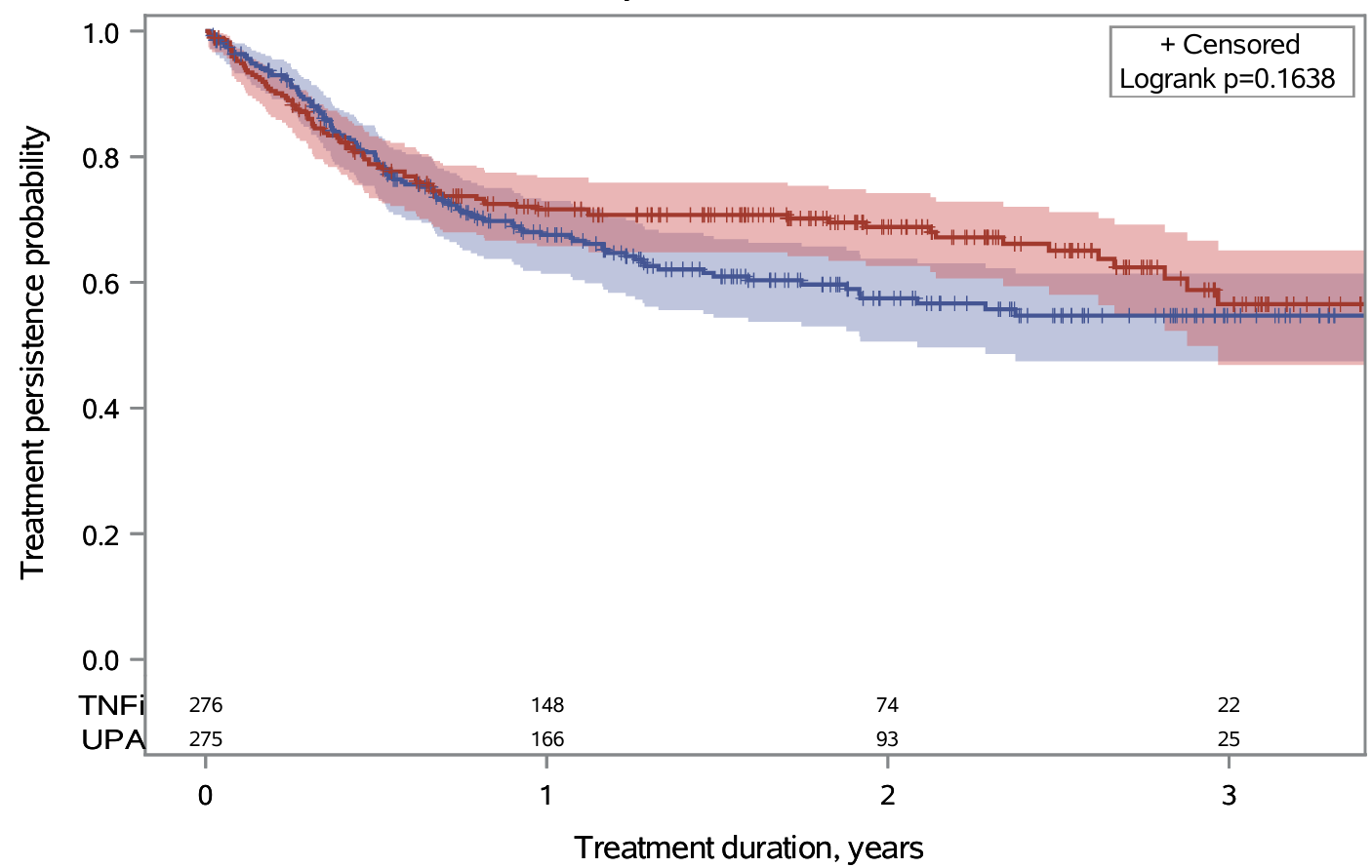

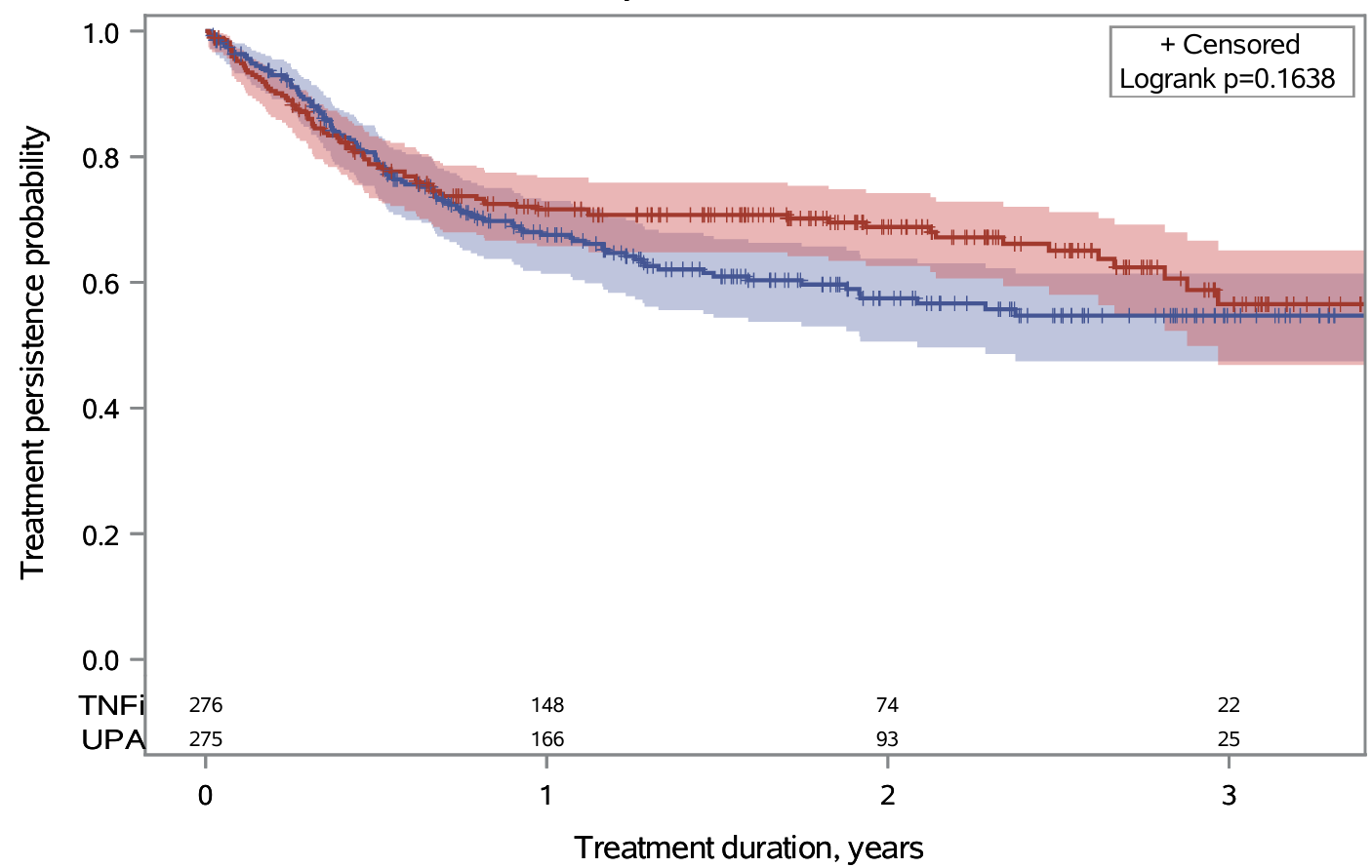

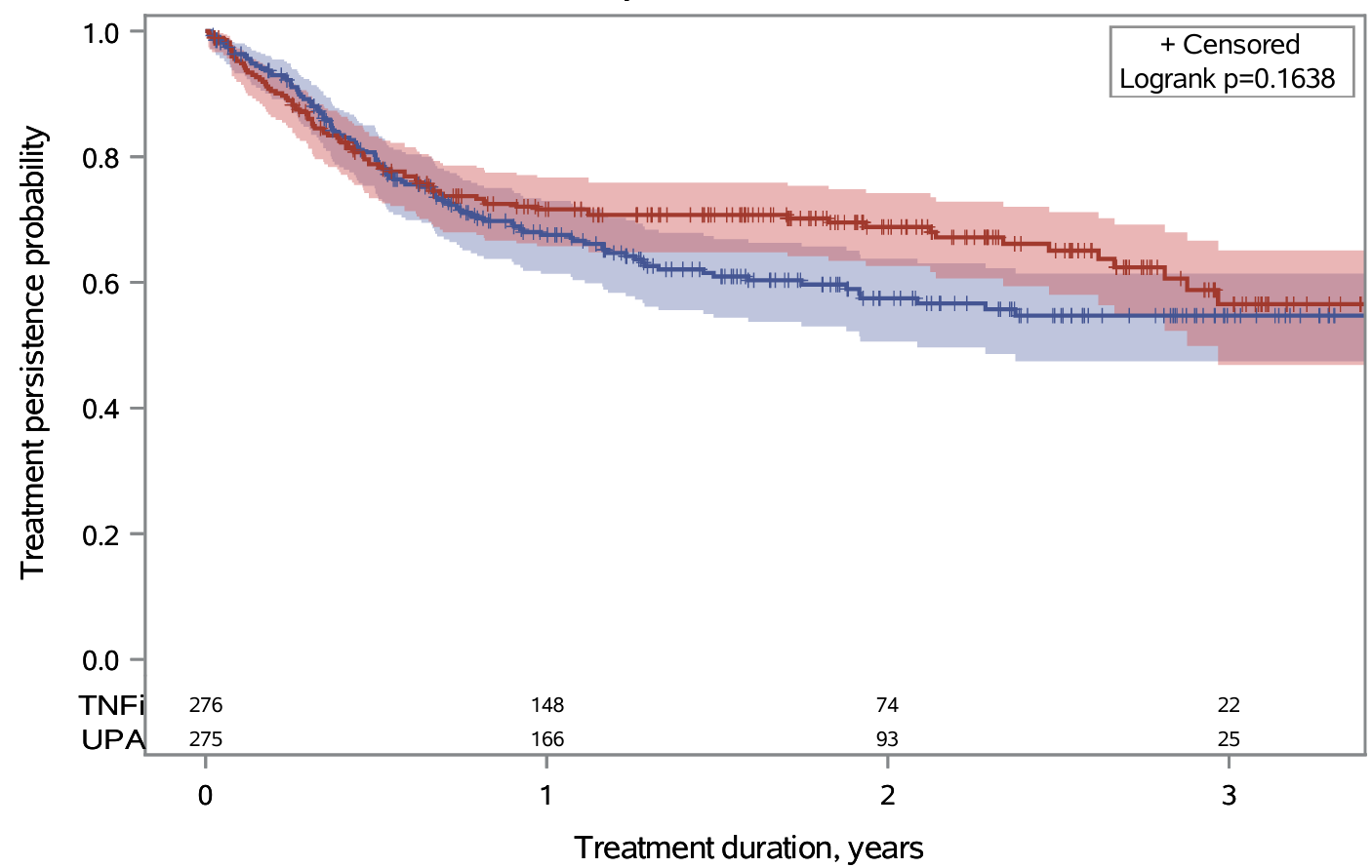


**A**

**B**

**C**

**D**


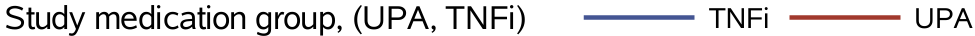


**Supplementary Figure S4.** **Comparison of the time to treatment discontinuation between TNFi and upadacitinib by non-TNFi previous treatment.** (**A**) Patients previously exposed to a non-TNFi; (**B**) patients previously exposed to a JAKi; (**C**) patients naïve to prior JAKi (first treatment or no previous JAKi); and (**D**) patients naïve to prior non-TNFi (first treatment or no previous non-TNFi). Shaded area represents 95% CI. JAKi, Janus kinase inhibitor; TNFi, tumour necrosis factor inhibitor; UPA, upadacitinib.
